# A Center-of-Pressure Guided Finger-Press Sensor for Cuffless Blood Pressure Estimation

**DOI:** 10.3390/s26175563

**Published:** 2026-09-01

**Authors:** Farhad Ali Irinel Gul, Dan Tudose

**Affiliations:** Department of Computer Science and Engineering, Faculty of Automatic Control and Computers, National University of Science and Technology POLITEHNICA Bucharest, 060042 Bucharest, Romania; farhad_ali.gul@upb.ro

**Keywords:** biofeedback, cuffless blood pressure monitoring, machine learning, multi-sensor systems, oscillometry, photoplethysmography (PPG), regression analysis

## Abstract

Cuffless blood pressure estimation using the finger-pressing method remains sensitive to improper finger centering and inconsistent contact force, which degrade the accuracy of the oscillometric envelope and PPG signal morphology. This paper details the development of a research prototype that integrates three force sensors and a photoplethysmograph (PPG) sensor to quantify the finger–device interaction. This system is intended as a pre-clinical research tool rather than a clinically validated medical device. We implement a weighted centroid algorithm for center of pressure (CoP) feedback to guide geometric centering, alongside a Hybrid Ridge Regression model to estimate the total contact force. The system was evaluated on a pre-clinical pilot cohort of 48 healthy participants (1274 recordings), comparing inflationary (ramp-up) and deflationary (ramp-down) interaction modalities. Force calibration achieved a mean absolute error (MAE) of 1.2 g, with hardware analysis confirming a limited zero-load baseline drift of −0.29% over 50 days. The best single-recording calibrated model achieved a mean absolute error (MAE) of 5.55 mmHg (systolic) and 5.20 mmHg (diastolic), with a mean error (ME) ± standard deviation (SD) of +0.50±7.25 and +1.19±6.47 mmHg, respectively, in this pilot cohort, demonstrating the feasibility of the three-point force-sensing design with CoP tracking.

## 1. Introduction

Cardiovascular diseases (CVDs) remain the leading cause of mortality globally [1], where hypertension is particularly dangerous due to its asymptomatic progression. Frequent and accurate monitoring of blood pressure is essential for early detection, allowing for early intervention before irreversible damage occurs. Currently, the clinical gold standard for measuring blood pressure is the sphygmomanometer with a brachial cuff. However, the mechanical occlusion required by this method causes user discomfort, making it unsuitable for continuous monitoring. Phenomena such as white coat hypertension where a user’s blood pressure rises due to the stress of the clinical setting, and masked hypertension often go undetected [2].

Recent advancements in multimodal biosensing have explored the integration of photoplethysmography with contact force measurements [3] to improve blood pressure estimation accuracy.

To systematically position our proposed solution against the current state of the art, we present a synthesis of our architectural decisions in Table 1. This matrix contrasts the standard approaches found in the literature with the specific design choices implemented in this study.

To address these limitations, this paper presents the design and evaluation of a high-fidelity finger BP monitor. By utilizing a non-invasive measurement method that eliminates the need for a cuff, comfort is improved for both users and healthcare providers. Using real-time visual guidance for applying pressure, users can operate the device without specialized medical training. This ease of use facilitates daily monitoring, allowing for the early prevention and management of health issues related to hypertension.

Unlike standard single-point force sensors, our system uses a rigid metal plate that rests on three force sensors positioned in a triangular formation. This hardware configuration, paired with a Hybrid Ridge Regression algorithm, allows for the calculation of the center of pressure (CoP) and the accurate estimation of applied force regardless of finger placement.

The primary contributions of this work are as follows:

- **Hardware Implementation:** We detail the complete realization of the device, from schematic design and PCB fabrication to the mechanical engineering of the metal force plates.
- **Precision Force Estimation:** We propose a hybrid regression model combining Linear Ridge Regression with quadratic correction. This approach mitigates mechanical cross-talk and sensor nonlinearity, achieving a mean absolute error (MAE) of 1.2 g while maintaining high positional stability.
- **Methodological Comparison:** We investigate the ergonomic and signal quality trade-offs between two user interaction protocols: the deflationary (ramp-down ↘) method versus the inflationary (ramp-up ↗) method.
- **Dataset Acquisition:** We compiled a multi-modal dataset comprising paired photoplethysmography (PPG) waveforms, force sensors, and reference cuff measurements across a group of 48 participants.
- **One-Point Cuff Personalization:** We designed and validated a machine learning pipeline (featuring an optimal 1D-CNN architecture) that bridges global feature extraction with localized user hemodynamics.

## 2. Hardware and Force Sensor Calibration

The device was independently developed in an academic laboratory by the first author under academic supervision. The work relies on public literature, published patents, and original experimental development.

### 2.1. Hardware Design

**Figure 1:**
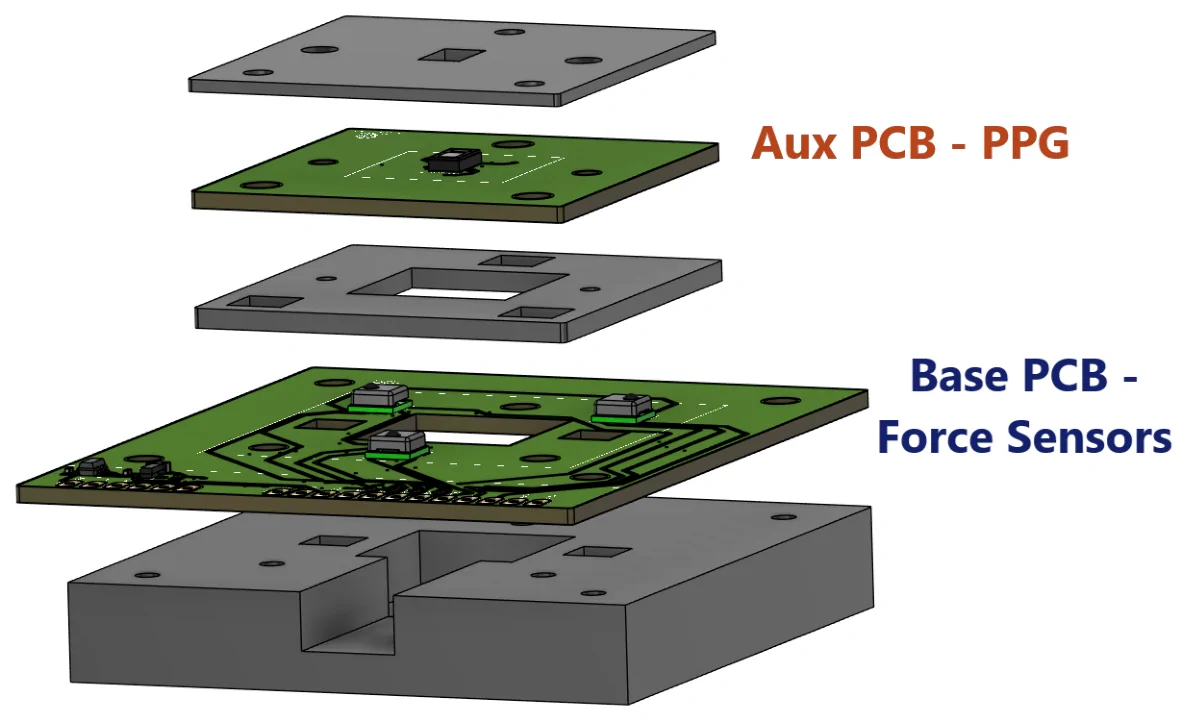
Cross-section of the hardware extension showing the multi-layered assembly of PCBs and metal plates.

To facilitate blood pressure measurement, we developed a specialized hardware extension based on our custom development board platform [7] consisting of a multi-layered assembly of two PCBs and three precision-engineered metal plates. This stack-up, designed using Autodesk Fusion 360 [8] and illustrated in Figure 1, is designed to provide a rigid interface for the force sensors while maintaining a compact form factor suitable for finger-pressing measurements.

For visual consistency and immediate cross-referencing with hardware schematics, multi-channel diagrams, and signal traces, a uniform color-coding scheme is maintained throughout the text, equations, and figures: *Base PCB* (dark blue), *Aux PCB* (dark red), the three tri-axial force channels *P*_1_ (blue), *P*_2_ (green), and *P*_3_ (orange), as well as the optical emission channels (red at 660 nm and infrared at 880 nm).

The primary circuit board, designated as the *Base PCB*, integrates three force sensors (FMAMSDXX015WCSC3 [9]), arranged in an equilateral triangle configuration. This layout was chosen to define a geometric plane, thereby enabling the calculation of the center of pressure (CoP). We specifically selected a three-sensor configuration because, geometrically, three non-collinear points uniquely define a plane, creating a *statically determinate* (isostatic) system that ensures constant contact and structural stability. In contrast, a four-sensor setup is statically indeterminate; slight manufacturing or assembly imperfections could cause the force plate to *wobble* (similar to a table with one short leg), leading to unpredictable load distribution and mechanical crosstalk. The top layer of the secondary board, the *Aux PCB*, is dedicated to the optical sensing module. We integrated the MAX30102 [10], a high-sensitivity pulse oximeter and heart-rate sensor. This module features internal LEDs emitting red (660 nm) and infrared (IR) (880 nm) light, alongside integrated photodetectors and low-noise electronics with ambient light rejection communicating with the microcontroller via the I2C bus [11], while the force telemetry is digitized and transmitted via the SPI protocol [12].

### 2.2. Center of Pressure (CoP) Estimation

To ensure optimal finger placement during measurement, we implemented a weighted centroid algorithm that calculates the instantaneous center of pressure (CoP). This feedback loop allows the system to guide the user to center their finger on the optical axis, minimizing artifacts caused by uneven pressure distribution. The geometric setup utilized for the CoP calculation involves three force sensors, $P_{1}$, $P_{2}$, and $P_{3}$, forming an equilateral triangle configuration. Each force sensor is positioned equidistant (approximately 17 mm) from the central MAX30102 optical sensor. This symmetry is important for ensuring uniform mechanical leverage around the measurement point.

To estimate the spatial position of the finger press, we first compute the active force components $f_{1}$, $f_{2}$, and $f_{3}$ by subtracting the baseline zero-offsets from the raw readings $p_{i}$:

(1) $$f_{i}=\max\left(0,p_{i}-{\mathrm{offset}}_{i}\right),\;i\in \left\{1,2,3\right\}$$

If the sum of these active forces ($f_{1}+f_{2}+f_{3}$) is below a minimum detection threshold of 50 units, the system registers no active contact ($X_{\mathrm{CoP}}=0.0,Y_{\mathrm{CoP}}=0.0$). Otherwise, the raw horizontal and vertical directional vectors are calculated based on the angular geometry of the sensor triangle:

(2) $$\begin{array}{cc} x_{\mathrm{vec}} & =0.775\cdot f_{2}-0.775\cdot f_{3} \end{array}$$

(3) $$\begin{array}{cc} y_{\mathrm{vec}} & =-1.0\cdot f_{1}+0.5\cdot f_{2}+0.5\cdot f_{3} \end{array}$$

The directional coefficients (−1.0, 0.5, and ±0.775) represent the scaled coordinate projections mapped to the GUI display workspace, where the horizontal scaling (±0.775 instead of the geometric ±0.866 derived from the cos(30°) projection of the equilateral configuration) was configured to calibrate the visual sensitivity of the centering interface. This empirical reduction in horizontal sensitivity balances visual feedback and limits rapid left–right oscillations on the display during active pressing.

The coordinates of the center of pressure $\left(X_{\mathrm{CoP}},Y_{\mathrm{CoP}}\right)$ are then obtained by normalizing these vectors by the total force and scaling them by a display scaling factor S=5.0 a.u. to map the coordinates onto the GUI’s circular target space (arbitrary units, a.u.):

(4) $$X_{\mathrm{CoP}}=\left(\frac{x_{\mathrm{vec}}}{f_{1}+f_{2}+f_{3}}\right)\cdot S,\;Y_{\mathrm{CoP}}=\left(\frac{y_{\mathrm{vec}}}{f_{1}+f_{2}+f_{3}}\right)\cdot S$$

**Figure 2:**
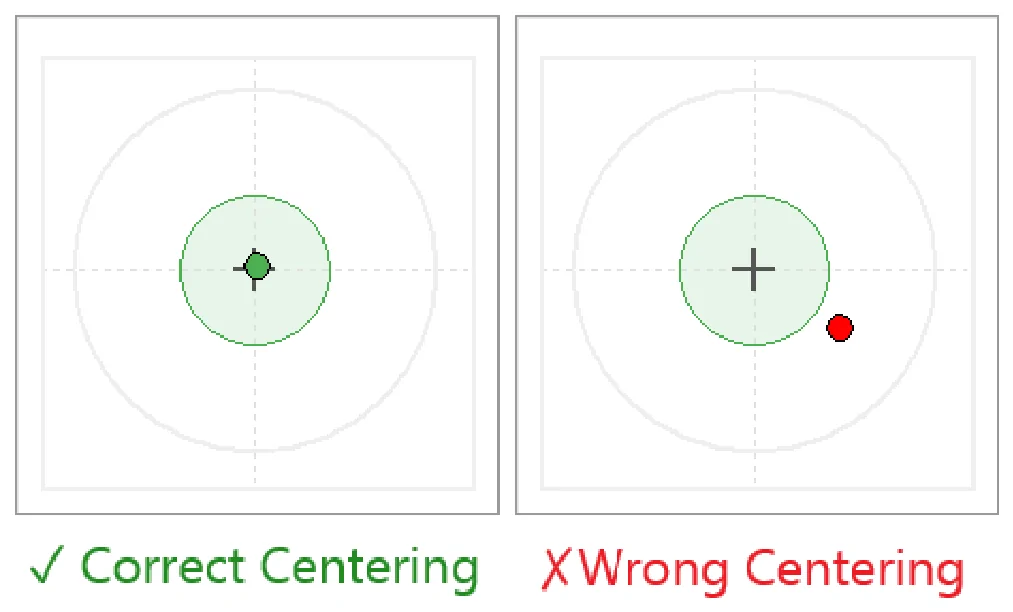
Visual guidance for finger placement. **Left:** Correct, perfectly centered application of force. **Right:** Off-center application, which may degrade measurement accuracy.

This real-time calculation drives the visual bullseye interface, guiding the user to minimize spatial centering error during the pressing cycle, as shown in Figure 2.

### 2.3. Force and Pressure Estimation Flow

To reconstruct the contact force and the corresponding pressure exerted by the user’s finger from this dataset, we developed a multi-stage physical conversion engine. This process maps the raw sensor telemetry to calibrated mass (grams), physical force (Newtons), and pressure (mmHg) using a Stage-1 linear extraction followed by a Stage-2 quadratic correction.

#### 2.3.1. Stage 1: Linear Ridge Extraction

First, the factory zero-load offsets (FACTORY_OFFSETS) are subtracted from the raw sensor values $p_{i}$ to yield the net sensor deflections $d_{i}$:

(5) $$d_{i}=p_{i}-{\mathrm{FACTORY}\text{\_}\mathrm{OFFSETS}}_{i},\;i\in \left\{1,2,3\right\}$$

Baseline zero-offsets ($p_{i,\mathrm{zero}}$) are automatically captured for each force sensor during an initial 10 s zero-load taring sequence prior to finger contact at program initialization and subtracted from raw sensor counts ($d_{i}=p_{i}-p_{i,\mathrm{zero}}$) to eliminate structural tare weight and sensor offset drift.

A base mass $M_{\mathrm{ridge}}$ (expressed in grams) is then extracted using regularized linear ridge weights ($w_{1},w_{2},w_{3}$) to ensure that the estimated mass remains position-invariant and stable even under off-center loading:

(6) $$M_{\mathrm{ridge}}=w_{1}d_{1}+w_{2}d_{2}+w_{3}d_{3}$$

#### 2.3.2. Stage 2: Non-Linear Quadratic Correction

To compensate for mechanical flexure and structural non-linearities of the metallic force plate, a global quadratic correction is applied to the extracted base mass:

(7) $$M_{\mathrm{corrected}}=A\cdot M_{\mathrm{ridge}}^{2}+B\cdot M_{\mathrm{ridge}}+C$$

where *A*, *B*, and *C* are the global polynomial coefficients.

The final corrected mass $M_{\mathrm{final}}$ is obtained by subtracting the dynamic zero-tare offset ($M_{\mathrm{tare}}$) captured during the initial zero-load sequence and multiplying by the calibration system gain ($G_{\mathrm{final}}$):

(8) $$M_{\mathrm{final}}=\left(M_{\mathrm{corrected}}-M_{\mathrm{tare}}\right)\cdot G_{\mathrm{final}}$$

where $M_{\mathrm{tare}}$ is the dynamic offset and $G_{\mathrm{final}}$ is the system calibration gain (set to unity 1.0 for this prototype, allowing for global scaling adjustments).

#### 2.3.3. Stage 3: Conversion to Physical Force and Pressure

The final mass $M_{\mathrm{final}}$ is converted into physical contact force *F* (in Newtons) and estimated finger contact pressure *P* (in mmHg) using the following relations:

- **Total Force (Newtons):**
  (9) $$F_{\mathrm{Newtons}}=\frac{M_{\mathrm{final}}}{1000}\cdot g$$
  where g=9.80665 m/s2 represents the standard acceleration due to gravity [13], and the divisor 1000 converts the mass from grams to kilograms.
- **Estimated Contact Pressure (mmHg):**
  (10) $$P_{\mathrm{mmHg}}=\left(\frac{M_{\mathrm{final}}}{\mathrm{Area}}\right)\cdot K_{\mathrm{mmHg}}$$

where area=1.45 cm2 represents the average effective finger–sensor contact area derived from mechanical fingertip models under typical pressing forces [14], consistent with baseline contact areas used in similar finger-pressing systems [4], and $K_{\mathrm{mmHg}}=0.735559$ is the conversion constant. We note that in the software implementation, a nominal area of 1.${\mathrm{1 cm}}^{2}$ was used for real-time processing. This constant scaling difference does not affect the predictive accuracy, as the machine learning models are scale-invariant. Neural network inputs are standardized (*Z*-score-normalized) prior to training, and tree-based splits scale proportionally, keeping the model optimization mathematically identical.

In this pilot prototype, the contact area is assumed to be constant, while in physical interactions the contact area varies dynamically with the applied pressure, assuming a constant area represents a baseline approximation necessary due to the absence of a dynamic contact-area imaging sensor. To evaluate the impact of this constant assumption, a sensitivity analysis was performed for a standard reference load of 200 g across the range of typical fingertip contact areas (1.0 to 1.${\mathrm{5 cm}}^{2}$): an area of 1.${\mathrm{0 cm}}^{2}$ yields an estimated pressure of 147.1 mmHg, which decreases to 133.7 mmHg for 1.${\mathrm{1 cm}}^{2}$ (a −9.1% change), to 101.5 mmHg for 1.${\mathrm{45 cm}}^{2}$ (a −31.0% change), and to 98.1 mmHg for 1.${\mathrm{5 cm}}^{2}$ (a −33.3% change). Because the estimated pressure scales inversely with the contact area (P∝1/Area), variations in finger size or contact geometry scale the absolute values uniformly. Due to soft-tissue damping, skin elasticity, and phalanx bone rigidity, surface pressure is non-uniformly transmitted through the tissue layer to the underlying digital artery ($P_{\mathrm{tissue}}=\gamma \cdot P_{\mathrm{surface}}$, where γ∈(0,1) represents the non-dimensional tissue pressure transmission coefficient, accounting for the mechanical damping and force distribution of soft tissue, skin, and underlying finger bone structure). Consequently, $P_{\mathrm{mmHg}}$ does not reflect the exact internal pressure exerted directly on the digital artery, but rather serves as a surrogate mechanical feature (a standardized external loading index) for downstream machine learning blood pressure prediction.

### 2.4. Force Calibration Dataset

To ensure high-fidelity force estimation and accurate blood pressure derivation, we conducted a calibration procedure using a set of standard reference masses. The calibration dataset encompassed a range of weights chosen to mimic the typical force exerted during finger pressing: 0 g, 50 g, 100 g, 120 g, 150 g, 200 g, 220 g, 250 g, 300 g, 320 g, 350 g, and 400 g. Each calibration session lasted for a fixed duration of 10 s with a sampling rate of 100 Hz, yielding a dataset of 1000 samples per file. For each reference mass class, two distinct acquisition trials were recorded: one during the *loading* phase (increasing from 0 g to 400 g) and one during the *unloading* phase (decreasing from 400 g to 0 g). This dual-phase approach was designed to characterize potential mechanical hysteresis in the sensor array or the metal plate. To minimize lever-arm errors, the script enforces a *center of pressure (CoP)* check.

The resulting dataset consists of 24 structured CSV files, representing a total of 24,000 telemetry records. To prepare the data for training, the raw readings of each file were filtered for outliers using a Z-score threshold of 3.0 and then averaged to produce a single representative feature vector ($p_{1},p_{2},p_{3}$) per trial. The regression models were trained on this consolidated training dataset of 24 samples (representing the loading and unloading phases across the 12 centered mass classes) to optimize the global calibration coefficients.

### 2.5. Regression Model Evaluation

We evaluated several regression architectures to map the sensor inputs to the target mass. A critical performance metric, beyond simple error minimization, was the model’s positional stability—the ability to report a consistent mass regardless of where the finger presses on the plate.

- **Linear Models (Standard and Linear Ridge):** These models assume a direct linear relationship ($F=\sum w_{i}x_{i}$). Testing revealed that standard Ordinary Least Squares (OLS) models exhibit low stability under positional variance, acting unpredictably when pressure is applied off-center. In contrast, linear ridge models limit extreme weights, yielding a balanced distribution across sensors and maintaining high stability.
- **Polynomial Models (Quadratic, Cubic, and Quadratic Ridge):** These models introduced higher-order terms ($x^{2},x^{3}$) to map the complex response curves. They proved to be practically unusable due to low stability; they essentially *overfit* the geometry, requiring the user to press exactly in the center. Any off-center pressure caused the calculated mass to drift significantly.

To demonstrate the accuracy of the force sensor calibration, we evaluated the performance of the models using the *mean absolute error* (MAE) metric, expressed in grams (lower is better ↓). We chose this metric because it provides a direct measure of the average deviation of the predictions from the actual applied force, while preserving the original unit of measurement of the physical system (grams). Unlike other metrics, such as *root mean square error* (RMSE), MAE does not disproportionately penalize outliers, thereby providing a more robust and accurate reflection of the sensor’s overall performance across the entire range of weights [15].

The training performance (fitting error) comparison of all evaluated methods on the centered calibration dataset is presented in Table 2.

The device achieved optimal performance using a standard Ordinary Least Squares (OLS) linear regression strictly under centered conditions. This baseline model yielded an exceptionally low training MAE of just 0.85 g. However, as subsequent independent validation testing revealed, this accuracy is not maintained under off-center loading, indicating low positional stability.

### 2.6. Proposed Hybrid Architecture

To resolve the conflict between stability, accuracy, and, most importantly, linearity, we developed a hybrid two-stage approach:

1. **Stage 1: Linear Ridge Extraction.** We utilize a strict *Linear Ridge* model to estimate a *base mass*. This ensures the reading remains position-invariant and stable [16].
2. **Stage 2: Non-Linear Correction.** We apply a global quadratic polynomial function to the output of Stage 1. This corrects the non-linear response of the sensors without re-introducing the geometric instability of individual sensor polynomials.

We opted for this hybrid two-stage architecture based on the following fundamental engineering considerations:

- **Stability and Multicollinearity Mitigation:** For Stage 1, we chose to utilize Linear Ridge regression instead of standard Ordinary Least Squares (OLS) to effectively manage multicollinearity between the sensors. By applying an L2 penalty to excessively large coefficients, the Ridge model forces a more balanced weight distribution across all three sensors ($P_{1},P_{2},P_{3}$). This solves the high variance associated with off-center finger placements and prevents any single sensor from dominating the prediction due to localized pressure spikes. Consequently, the extracted base mass remains geometrically stable and robust to user positioning errors before the non-linear correction is applied.
- **Separation of Concerns:** This approach cleanly decouples the physical phenomena. Stage 1 isolates the ideal linear response of the sensor array, while Stage 2 specifically addresses the physical mechanics (e.g., the inherent bending or mechanical nonlinearity of the metal plate).
- **Generalization vs. Overfitting:** By dividing the problem into a regularized linear extraction followed by a controlled global polynomial correction, the model is strictly constrained. This drastically reduces the degrees of freedom compared to an independent polynomial regression, thereby minimizing the risk of overfitting to localized noise or pressure spikes.

The training performance (fitting error) results obtained from running the Hybrid Approach on the centered calibration dataset are presented in Table 3.

As observed in Table 3, the proposed hybrid architecture achieves a mean absolute error (MAE) of 1.20 g. Although this value is marginally higher than the baseline OLS model (MAE = 0.85 g), the difference of merely 0.35 g is practically negligible in the context of finger-based blood pressure measurements.

### 2.7. Force Calibration Positional Stability Validation

**Figure 3:**
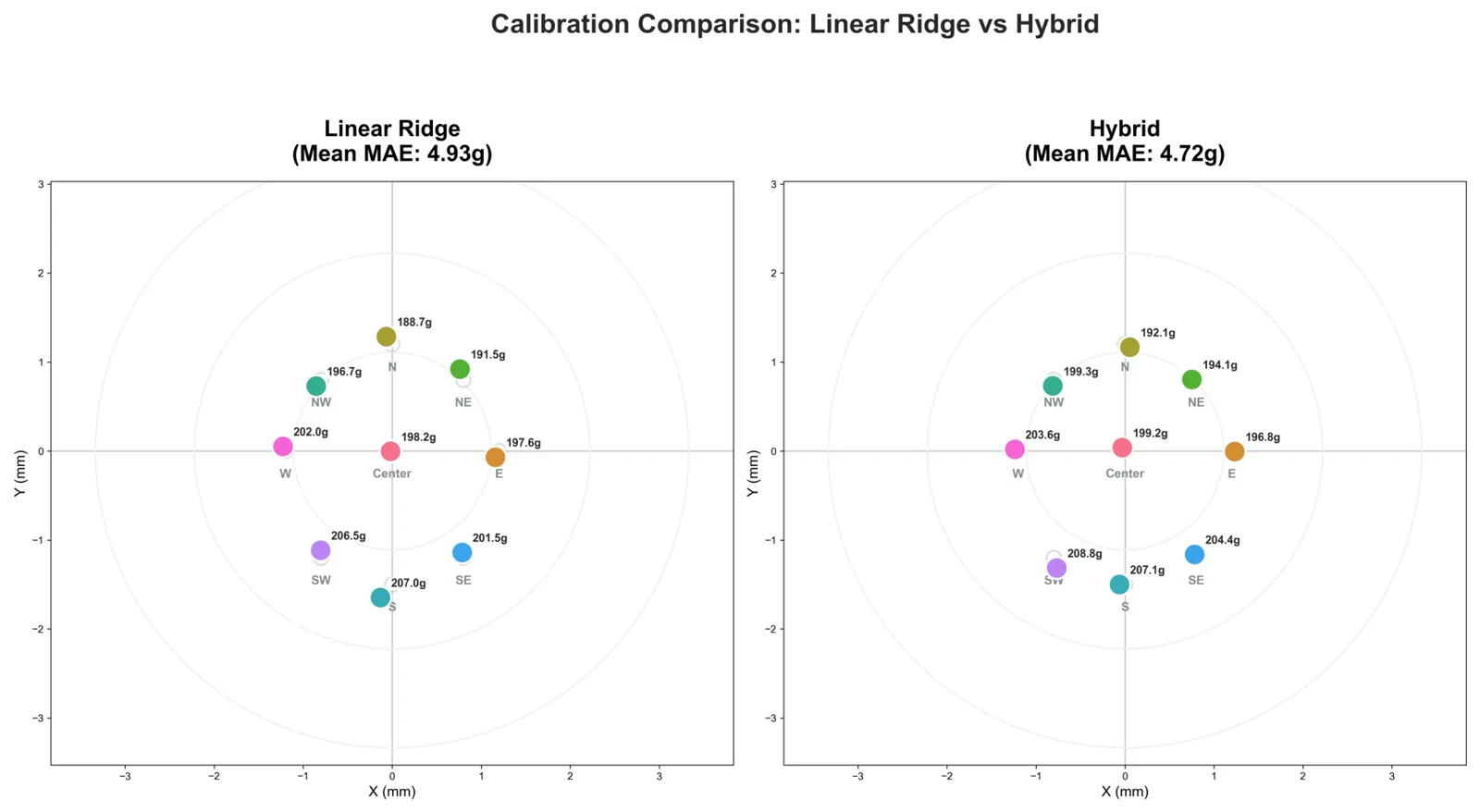
Spatial distribution of mean absolute error (MAE) across a 9-point validation grid using a 200 g reference mass. The hybrid method performs slightly better than a ridge regression on non-centered data points.

To evaluate the spatial robustness of the force estimation to off-center presses (positional stability), a separate validation dataset was collected. A standard reference mass of 200 g was positioned at 9 different spatial coordinates on the metal force plate (center, four cardinal edges, and four diagonal corners). The spatial distribution of the resulting MAE is plotted in Figure 3.

This spatial evaluation explains the difference between the training errors reported in Table 2 and Table 3 (which were evaluated strictly on the centered training set) and the generalization performance on the independent spatial test set. To quantitatively evaluate this positional stability, Table 4 reports the mean absolute error (MAE) obtained across all 9 grid points under the 200 g reference load.

While the OLS baseline model drifts significantly when the load is applied off-center (achieving a high test MAE of 32.96 g), the regularized Ridge and Hybrid models demonstrate high positional stability on this independent test set, achieving test MAE values of 4.93 g and 4.72 g, respectively.

## 3. Data Collection Protocol and Dataset

### 3.1. Collection Protocol

To validate the accuracy of the device across different physiological states, we devised a rigorous data collection protocol. The experimental design, protocol parameters, and human subject data acquisition workflows were formally reviewed and approved by the Ethics Committee for Research and Academic Integrity of the Faculty of Automatic Control and Computer Science at the National University of Science and Technology POLITEHNICA Bucharest (registration no. AC 835/04.03.2023, decision no. AC 2/04.03.2023).

Written informed consent was obtained from all participants prior to their involvement in the study. Each participant underwent a structured session lasting approximately 45–50 min. To ensure physiological stability and prevent transient cardiovascular spikes, participants were required to adhere to the following constraints for at least 30 min prior to their arrival:

- No caffeine consumption;
- No nicotine use;
- No alcohol intake;
- No moderate or vigorous physical exercise.

Upon arrival, participants were required to rest in a relaxed, seated position for 20 min. During this time and throughout the session, subjects maintained a standardized posture: back fully supported by the chair, feet flat on the floor, and legs uncrossed, with both the reference arm and the hand operating the sensor supported at heart level. Strict silence and immobility were enforced during all active measurement windows.

Prior to the measurement phase, demographic and physical characteristics were collected to facilitate stratified post hoc analysis. All personal identifiers (such as names and email addresses) were stored in a secure registry separate from the experimental telemetry dataset, with each participant assigned a unique random ID to ensure confidentiality:

- **Identity:** participant ID (anonymized).
- **Physical:** age, gender, height (cm), weight (kg).
- **Biometrics:** skin tone (Fitzpatrick scale 1–6), finger size (mm).
- **Medical History:** known hypertension (yes/no), smoking status (yes/no), known arrhythmias (yes/no).

### 3.2. Session Architecture

The data collection session was systematically divided into sequential phases, designed to establish a baseline and evaluate both actuation methodologies. Prior to formal data logging, a brief device familiarization period (5–10 repetitions) allowed users to practice GUI interaction and pressure control. Data from these practice runs were explicitly flagged as testing and excluded from the validation dataset to prevent bias. Each recording has a sampling rate of 200 Hz. The operational details are summarized in Table 5.

The first phase (calibration) was designed to quantify the systematic inter-arm blood pressure difference ($\Delta_{L-R}$) for each subject. To ensure that the contralateral reference measurement serves as a valid representation of the cardiovascular pressure on the finger-pressing side, subjects were screened using a strict systolic blood pressure (SBP) difference threshold of 5 mmHg for exclusion. In this study, all screened participants fell below this threshold, exhibiting minimal inter-arm variation. Consequently, no participant was excluded from the cohort. The arm with the consistently higher SBP was selected as the reference arm, and the contralateral hand was used to operate the finger BP monitor.

### 3.3. Reference Blood Pressure Measurement Protocol

To establish a high-fidelity reference BP measurement for the machine learning pipeline, reference blood pressure measurements were acquired using a commercial automated oscillometric upper-arm sphygmomanometer (Sendo One, manufactured by HONSUN (NANTONG) Co., Ltd., Nantong, China) equipped with a standard upper-arm cuff (medium size, 22–32 cm arm circumference). This reference device is CE-certified and manufactured in compliance with international safety and performance standards for non-invasive sphygmomanometers. To eliminate human hearing bias and ensure objective readings, measurements relied exclusively on the automated digital outputs of the device, and no manual auscultatory observers were employed.

Our study utilized a simultaneous acquisition paradigm to synchronize the reference device with the finger-pressing sensor. For each recording, the reference cuff was placed on the arm selected during the arm-dominance calibration phase (i.e., the arm with consistently higher SBP), while the finger BP monitor was operated by the contralateral hand. Synchronization was achieved by initiating the finger-pressing protocol (lasting approximately 15–20 s) concurrently with the start of the reference cuff’s inflation cycle. This concurrent execution ensures that the reference BP measurements and the multi-modal finger signals (PPG and force) capture approximately contemporaneous cardiovascular conditions. A strict 15–30 s rest interval was enforced between consecutive repetitions. This interval was selected as a practical compromise to allow partial recovery of local hemodynamics and venous return between repetitions, while preventing prolonged venous pooling and maintaining participant focus during the session.

The contralateral configuration was mandatory. Placing the reference cuff on the same arm as the finger-pressing sensor would cause arterial occlusion during cuff inflation, completely corrupting the PPG waveform. The 15–30 s interval was selected as a trade-off to prevent venous congestion from repeated cuff inflations while keeping the overall session duration within tolerable limits for participants.

### 3.4. Dataset Characteristics

The baseline demographic, anthropometric, and hemodynamic characteristics of the study population (N=48) are summarized in Table 6. The group exclusively comprised young adults with no known diagnosis of hypertension (mean age 20.7±0.7 years, range 19–22), representing a highly controlled demographic baseline, while the cohort does not include patients with a chronic hypertensive diagnosis, individual hemodynamic recordings captured instances of transiently elevated blood pressure (reaching up to 155 mmHg systolic). These transient values represent situational fluctuations in healthy normotensive individuals and do not reflect clinical hypertension. Hence, the current model’s performance should not be assumed to generalize to patients with chronic hypertensive conditions.

**Figure 4:**
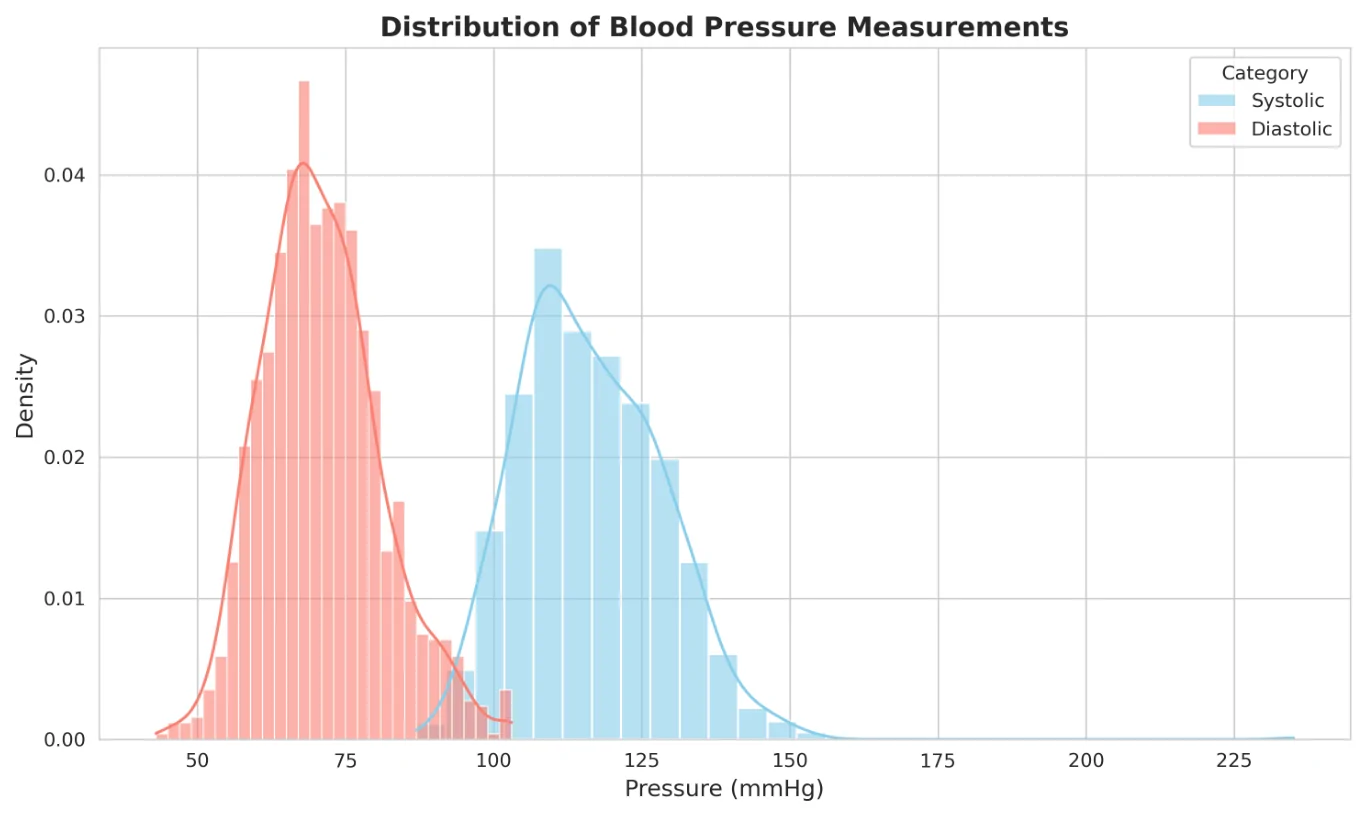
Density distribution of the collected reference blood pressure measurements.

Figure 4 shows the distribution of blood pressure. The peak diastolic blood pressure is approximately 70 mmHg, and the peak systolic blood pressure is 115 mmHg, values that are consistent with the expected resting blood pressure values for a group of healthy young adults.

The distribution of the collected telemetry across standardized clinical blood pressure categories is detailed in Table 7.

As shown in Table 7, the dataset exhibits a strong central concentration around normotensive values (56.7% systolic, 71.0% diastolic). In global machine learning models, this class imbalance induces a regression-to-the-mean effect, where predictions tend to pull toward the population mean. Consequently, uncalibrated global models exhibit proportional bias—overestimating hypotensive values (<100 mmHg) and underestimating hypertensive values (>140 mmHg). The localized one-point cuff calibration (N=1) successfully mitigates this limitation by shifting the subject-specific baseline, transforming the sloped proportional error into a zero-centered homoscedastic error distribution.

### 3.5. Dataset Cleanup and Signal Quality Index (SQI)

To ensure high signal fidelity, a signal quality index (SQI) and preprocessing pipeline were implemented. Initially, the raw digital force telemetry was converted into absolute pressure (mmHg) via a physics-based calibration engine, which integrated dynamic zeroing offsets and approximated fingertip morphometry. We retained only the 45–180 mmHg range, preprocessed the signal using a zero-phase second-order Butterworth bandpass filter, and excluded recordings that had a mean deviation exceeding 15 mmHg, did not correlate with the specified trend (deflating or inflating), or had fewer than eight extracted pulses. As can be seen in Table 8, of the initial 1291 captured recordings, 1274 (98.7%) met these rigorous quality standards and were retained for model training. Only 17 recordings (1.3%) were excluded due to excessive motion, trend instability, physiological out-of-bounds anomalies, or insufficient pulse morphology.

### 3.6. Ramp-Down (Deflating) vs. Ramp-Up (Inflating) Biomechanical Stability

In both modalities, users receive real-time visual feedback to maintain their optimal center of pressure (CoP), as detailed in the program flow architecture. The objective of this section is to analyze the high-frequency biomechanical deviation (sampled at 200 Hz) of the user’s applied force relative to the optimal CoP along the horizontal (OX) and vertical (OY) axes, comparing the ramp-down (↘) and ramp-up (↗) phases.

To accurately quantify this mechanical instability, we report the mean absolute deviation (MAD) rather than the standard arithmetic mean. MAD correctly characterizes the true magnitude of physical displacement; by taking the absolute value of all deviations, it prevents positive and negative directional ‘wiggles’ from canceling each other out.

Table 9 presents the aggregated MAD for the OX (left–right) and OY (up–down) axes, alongside the combined Euclidean error and total sample size across the dataset.

Under the current experimental protocol, lower center of pressure (CoP) deviation was observed during the ramp-up (↗) modality compared to ramp-down (↘). Specifically, the vertical OY error was lower during ramp-up (0.243±0.211 a.u.) than during ramp-down (0.313±0.225 a.u.). This observed association aligns with the physiological hypothesis that active muscle engagement during progressive force application may provide better digital stabilization (‘muscle anchoring’) than controlled relaxation during force release, which may be more susceptible to neuromuscular tremor or micro-slippage. However, this observed difference cannot be interpreted as a proven intrinsic physiological advantage due to the fixed order of protocol execution.

Beyond spatial stability, several plausible physiological mechanisms may contribute to or interact with the observed protocol differences under progressive versus regressive force loading:

- **Plausible Viscoelastic Tissue Hysteresis:** Fingertip soft tissue exhibits non-linear viscoelasticity. During progressive force application (ramp-up), tissue compression follows an active loading trajectory, whereas during force release (ramp-down), viscoelastic stress relaxation may cause tissue pressure response to lag behind mechanical force reduction, potentially introducing mechanical hysteresis as a candidate explanatory factor.
- **Plausible Post-Occlusive Hemodynamic Effects:** Because ramp-down actuation immediately follows peak compression (∼180 mmHg) when distal digital vessels are transiently occluded, transient post-occlusive reactive hyperemia and venous blood pooling could alter the optical DC baseline and distort dicrotic notch morphology during release. In contrast, progressive loading during ramp-up starts from an unoccluded baseline, offering a distinct physiological starting state.

*Methodological Confounding and Order Effects:* While active flexor muscle anchoring offers a plausible physiological explanation for the lower CoP deviation observed during ramp-up, a key methodological limitation must be emphasized: all participants performed the ramp-down protocol prior to the ramp-up protocol in a fixed acquisition sequence. Consequently, the lower CoP deviation observed during ramp-up represents an association under the current protocol that may be partially or substantially attributable to order effects, such as user learning, familiarization with the real-time visual guidance interface over consecutive trials, or motor adaptation, rather than an intrinsic biomechanical advantage of the inflationary protocol. Counterbalanced or randomized acquisition protocols in future studies are required to isolate true causal biomechanical mechanisms.

**Figure 5:**
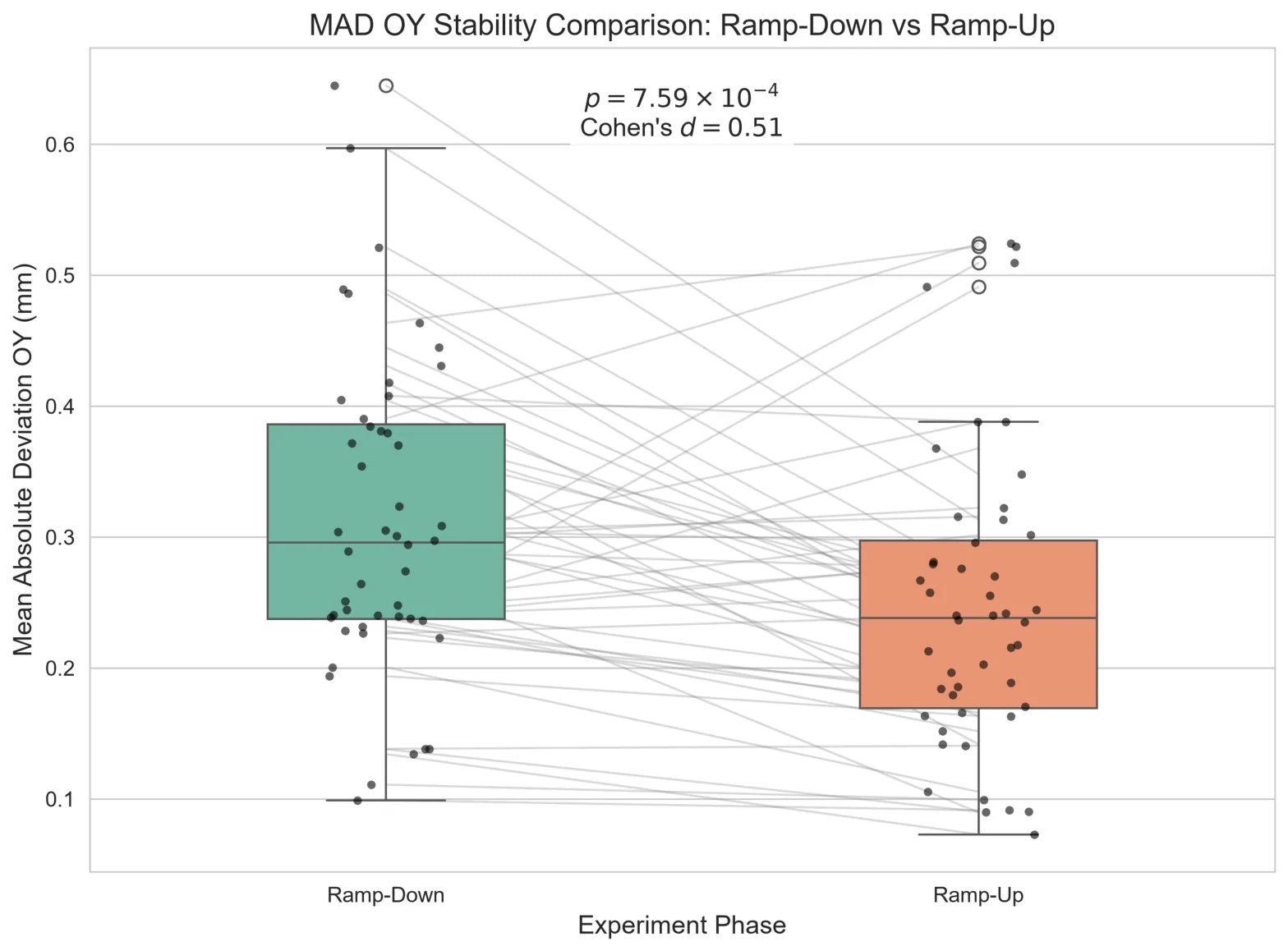
Paired comparison (slopegraph) of the mean absolute deviation (MAD) for the center of pressure across all 48 subjects.

Figure 5 visually maps the paired trajectory for each subject. The statistical analysis demonstrates a reduction in CoP deviation during the ramp-up (↗) phase relative to ramp-down (↘) within this fixed sequence. Specifically, along the primary OY axis, the ramp-up (↗) protocol yielded a reduction in CoP deviation with a *p*-value of $7.59\times 10^{-4}$ using the Wilcoxon signed-rank test [17]. In practical terms, this suggests a less than 0.1% probability that the observed improvement is attributable to random variance. The standardized effect size was quantified using Cohen’s *d* for paired samples, yielding a moderate effect of 0.5149 according to Cohen’s effect size scale [18]. This trend was consistent in the combined MAD spatial analysis ($p=1.33\times 10^{-3}$, d=0.4690).

### 3.7. Temporal Force Sensor Drift Characterization

**Figure 6:**
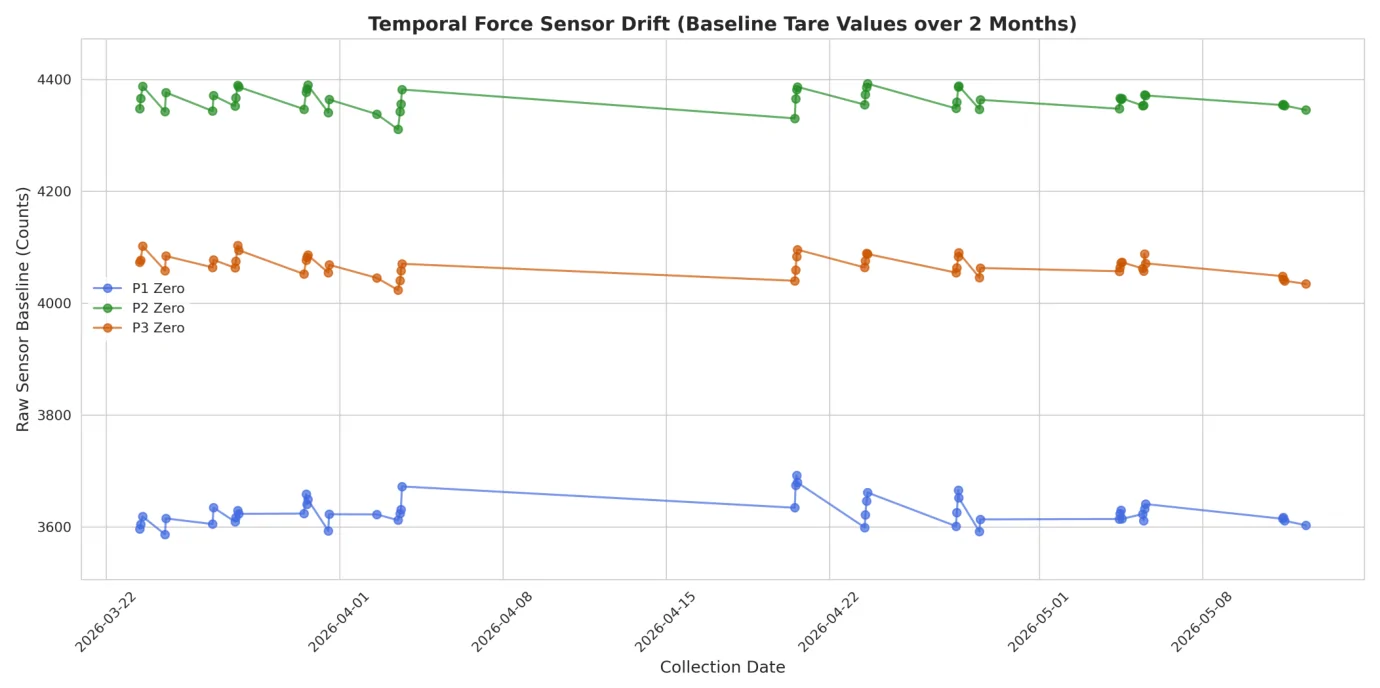
Longitudinal characterization of zero-load baseline drift across the three force sensors over successive measurements.

To evaluate the mechanical and electrical stability of the hardware interface over the duration of the data collection protocol, the zero-load baseline values (p1_zero, p2_zero, and p3_zero) were extracted from the metadata of each reference recording. These values represent the raw sensor counts captured at the initiation of each session, immediately prior to any user actuation or applied pressure. The longitudinal temporal drift of these baselines across the dataset is illustrated in Figure 6.

Figure 6 shows that while the sensors exhibit occasional transient deviations from their initial baselines, all three force sensors exhibit a highly synchronous and correlated phase shift when these offsets occur. Such synchronized deviations are indicative of external environmental or operational artifacts rather than independent piezoresistive hardware degradation. Most commonly, the user inadvertently rests a finger on the sensor plate prior to initiating the recording. To strictly mitigate these transient errors and ensure an accurate force-to-pressure conversion, the system implements a dynamic zeroing (taring) protocol at each program initialization. This ensures a continuously updated and corrected baseline for every individual measurement.

Table 10 presents comparative statistics for the cumulative zero-load baselines (the sum of p1_zero, p2_zero, and p3_zero) between the first and last day of the 50-day data collection period.

The baseline analysis yields an initial total count of 12,017.7 and a final total count of 11,982.5, resulting in an absolute net drift of merely −35.2 counts. Normalized against the initial values, this equates to a percentage net drift of −0.29% over the entire 50-day study duration.

While the software-level dynamic taring mechanism is used to compensate for short-term offset variations, the underlying hardware components exhibited mechanical and electrical stability over the 50-day evaluation period. A net drift of −0.29% indicates that the force-sensing platform showed limited zero-load baseline drift over nearly two months of use.

## 4. Blood Pressure Results and Analysis

### 4.1. Data Filtering and Extraction of Pulse Morphological Data

The derivation of blood pressure parameters from the raw sensor telemetry involves a multi-stage signal processing chain. The pipeline is designed to isolate the pulsatile arterial signal from mechanical noise and reconstruct the oscillometric envelope using a physiological model.

#### 4.1.1. Preprocessing and Signal Separation

The raw PPG signal is sampled at 200 Hz. To eliminate initialization artifacts, the first 100 samples of every acquisition window are discarded. The remaining signal is decomposed into its constituent AC and DC components using a second-order Butterworth filter [19]:

1. **DC Component (Low-Pass < 0.5 Hz):** Represents the baseline blood volume and the static pressure applied to the tissue. This provides a measure of how compressed the tissue is at any given moment.
2. **AC Component (Band-Pass 0.5–8.0 Hz):** Isolates the cardiac pulsatile wave. The 8.0 Hz cutoff is selected to preserve the morphological integrity of the dicrotic notch, which contains essential information regarding vascular resistance.

The modulation of these AC and DC components during finger pressing is governed by the net transmural pressure ($P_{\mathrm{tr}}=P_{\mathrm{art}}-P_{\mathrm{tissue}}$) across the digital arterial wall [5,20]. Under low applied pressure ($P_{\mathrm{tissue}}<\mathrm{DBP}$, under-loaded regime), $P_{\mathrm{tr}}>0$ and the arterial wall remains constrained by internal pressure, yielding lower PPG AC amplitude ($A_{\mathrm{norm}}$) [21]. As applied pressure approaches mean arterial pressure ($P_{\mathrm{tissue}}\approx \mathrm{MAP}$, unloaded regime), net transmural pressure approaches zero ($P_{\mathrm{tr}}\approx 0$). At zero transmural pressure, vascular wall compliance reaches its maximum, resulting in maximum pulsatile expansion and peak $A_{\mathrm{norm}}$ amplitude [4,20]. When applied pressure exceeds SBP ($P_{\mathrm{tissue}}>\mathrm{SBP}$, over-loaded regime), $P_{\mathrm{tr}}<0$, causing transient arterial collapse during diastole and severe attenuation of the AC pulse morphology.

#### 4.1.2. Adaptive Heartbeat Segmentation

To identify individual heartbeats within the AC signal, we implemented an adaptive peak detection algorithm. A valid heartbeat is registered only if the local peak prominence exceeds a dynamic threshold of 0.2σ, where σ represents the standard deviation of the AC signal within the given temporal window. This adaptive thresholding mechanism effectively suppresses low-amplitude noise and motion artifacts.

Heartbeat boundaries are defined between consecutive systolic peaks $\left[start_{i},end_{i}\right]=\left[{\mathrm{peak}}_{i},{\mathrm{peak}}_{i+1}\right]$. Peak detection enforces a minimum inter-peak distance constraint of $f_{s}/2.5=80$ samples (0.4 s, setting a maximum heart rate limit of 150 bpm) and cross-references peak indices between the infrared (IR) and red optical channels to ensure stable segmentation.

#### 4.1.3. Feature Normalization and Quality Control

For every detected heartbeat, we compute the normalized pulse amplitude ($A_{norm}$):

(11) $$A_{norm}=\left(\frac{AC_{peak-to-peak}}{DC_{avg}}\right)\times 1000$$

We utilize the AC/DC ratio to cancel out mechanical artifacts because the absolute strength of the PPG signal varies significantly with contact pressure (optical coupling). We apply a scaling factor of 1000 to convert the ratio into a manageable index. Here, $AC_{\mathrm{peak-to-peak}}$ is calculated as $\max\left(AC\left[start_{i}:end_{i}\right]\right)-\min\left(AC\left[start_{i}:end_{i}\right]\right)$ over the segment. Crucially, $DC_{\mathrm{avg}}$ is calculated locally per heartbeat by taking the mean of the lowpass-filtered baseline (<0.5 Hz) over that specific beat window $\left[start_{i},end_{i}\right]$ ($\frac{1}{end_{i}-start_{i}}\sum_{k=start_{i}}^{end_{i}-1}DC\left[k\right]$), rather than over a global window or the full recording, ensuring it dynamically tracks localized pressing force shifts.

**Noisy and Incomplete Beat Filtering:** The first 400 samples (2.0 s) of each recording are discarded to remove sensor placement transients. Pulse segments shorter than 10 samples (<50 ms) or with mean pressure outside the physiological range ($40<P_{\mathrm{mean}}<200$ mmHg) are rejected. Furthermore, beats are excluded if inter-channel optical correlation is low (pulse_corr < 0.4, motion artifacts) or spatial force imbalance across the three load sensors is high (imbalance >0.25, off-center pressing).

**Clinical Guidance Rejection:** We enforce a strict pressure-compliance filter to any recording where the applied pressure deviates by an average of >15.0 mmHg from the target guidance ramp is discarded from the dataset.

#### 4.1.4. Machine Learning Feature Engineering

While classical oscillometry relies on fitting a skewed Gaussian model to the $A_{norm}$ (pulse amplitude) envelope to determine mean arterial pressure (MAP) [20,22], our framework bypasses this intermediate envelope reconstruction by feeding the individual heartbeat morphology directly into machine learning architectures. The final systolic and diastolic values are derived via ML models (Random Forest, XGBoost, Multi-Layer Perceptron, and 1D-CNN).

The MAX30102 sensor module integrates dual-wavelength emission (infrared at 880 nm and red at 660 nm). In our architecture, the infrared (IR) channel is designated as the primary optical signal for waveform morphological analysis, 1D-CNN inputs, and primary feature extraction (norm_amp). Infrared light was selected as the primary channel due to its deeper optical penetration into the subcutaneous tissue layer containing the digital arterial bed, higher pulsatile signal-to-noise ratio (SNR) under pressing load, and reduced sensitivity to surface skin pigmentation. The red channel is integrated as a secondary signal, utilized to capture multi-wavelength absorption ratio dynamics (red_amp) and compute the dual-wavelength cross-correlation coefficient (pulse_corr), which acts as a signal quality index (SQI >0.4) to reject motion artifacts.

For the classical machine learning models (XGBoost [23], Random Forest [24], MLP [25]), we extract 17 hand-crafted features from every valid heartbeat segment. These features, summarized in Table 11, characterize the temporal, morphological, and physical pressure aspects of the individual pulse [21]. Out of the 17 pulse-level parameters summarized in Table 11, pulse_corr is used exclusively as a signal quality index (SQI) filter and confidence weighting metric ($W_{i}$), while the remaining 16 parameters are passed directly as input features to the classical regression models.

#### 4.1.5. Pulse Filtering and Record-Level Aggregation

To prevent noisy or corrupted pulses from biasing the predictions, we enforce a strict pulse-level quality filter. A heartbeat is retained for prediction only if the correlation between the IR and red channels is high (pulse_corr >0.4, acting as an optical signal quality index to filter motion artifacts) and the spatial pressing imbalance is low (imbalance <0.25, ensuring the finger remains geometrically centered within a 25% spatial force deviation threshold to minimize lever-arm errors).

For each remaining valid heartbeat *i*, a confidence weight $W_{i}$ is dynamically calculated:

(12) $$W_{i}=\frac{\mathrm{pulse}\text{\_}{\mathrm{corr}}_{i}}{{\mathrm{imbalance}}_{i}+0.01}$$

The final record-level blood pressure prediction ($P_{\mathrm{record}}$) is obtained by aggregating the individual beat-level predictions ($P_{\mathrm{beat},i}$) using a weighted average based on the confidence weights:

(13) $$P_{\mathrm{record}}=\frac{\sum_{i=1}^{M}W_{i}\cdot P_{\mathrm{beat},i}}{\sum_{i=1}^{M}W_{i}}$$

where *M* represents the total number of valid heartbeats in the recording.

#### 4.1.6. Machine Learning Architectures and Hyperparameters

The classical machine learning architectures (XGBoost, Random Forest, and Multi-Layer Perceptron) were trained using standardized hyperparameters. The MLP model utilized standard scaling (*Z*-score normalization) on the 17 input features prior to training, whereas the tree-based models (XGBoost and Random Forest) operated on the raw extracted features, utilizing the computed confidence weights ($W_{i}$) during the optimization phase. The exact hyperparameter settings for these architectures are detailed in Table 12.

*Input Representation and Model Performance:* A key distinction between the evaluated architectures is the input representation: while classical models (XGBoost, Random Forest, MLP) operate on the 16 hand-crafted scalar features (with pulse_corr used for quality filtering and weighting), the 1D-CNN receives the raw 128-point resampled 1D pulsatile PPG time-series waveform concatenated with mean pressure. In this pilot evaluation, the 1D-CNN achieved numerically superior predictive performance across the evaluated dataset.

*Feature Selection and Processing:* The 17 features were selected based on the clinical PPG literature [19,21] to capture applied contact pressure, multi-sensor spatial balance, optical pulse morphology, and derivative dynamics (VPG/APG). For model training, tree-based algorithms consume raw features weighted by pulse confidence ($W_{i}$), while neural networks (MLP) utilize *Z*-score-standardized feature vectors.

The proposed 1D-CNN [26] is designed to extract deep morphological features directly from the pulse waveform while incorporating the static physical context of the applied pressure. The architecture, detailed in Table 13, consists of a temporal feature extraction branch followed by a multi-modal fusion layer.

The waveform branch utilizes two stages of convolutional and max-pooling layers to reduce dimensionality and identify shift-invariant arterial signatures (such as the dicrotic notch and systolic slope). The resulting 960-dimensional temporal feature vector is concatenated with the scaled mean_p scalar. This combined representation is processed by a 32-unit dense layer with ReLU activation to learn the non-linear relationship between pulse morphology, contact pressure, and blood pressure. The model was trained using the Adam optimizer with a mean absolute error (MAE) loss function.

### 4.2. Statistical Analysis and Uncertainty Quantification

To quantify the uncertainty of the performance metrics and align with numerical clinical validation benchmarks, 95% confidence intervals (CIs) were calculated for the mean error (ME), standard deviation (SD), and mean absolute error (MAE) of the optimal 1D-CNN architecture. For the ME, we used the standard parametric approach ($ME\pm 1.96\times (SD/\sqrt{N})$), where *N* represents the total number of recordings. For the MAE and SD, which may follow non-normal distributions, we employed a bootstrap method with 1000 resamples to determine the 2.5th and 97.5th percentiles. To evaluate the system’s performance at the participant level, we report per-subject error metrics in addition to the global per-recording results.

To address the non-independence of repeated measurements, acknowledging that the 1274 recordings originate from a cohort of only 48 participants and should not be treated as completely independent observations, we leverage the properties of the localized calibration. Because the one-point calibration (N=1) anchors the predictions to each subject’s baseline, the systematic inter-subject offset bias is minimized, centering the subject-specific errors close to zero. Under these conditions, the pooled standard deviation converges to the repeated-measures standard deviation proposed by Bland and Altman [27], ensuring that the computed limits of agreement (LoAs) remain statistically valid. The normality of the distributions was assessed using the Shapiro–Wilk test [28]. For the CoP spatial stability comparison, statistical significance was evaluated using a paired Wilcoxon signed-rank test aggregated at the subject level.

### 4.3. Global Cross-Fold Training (Universal Generalization)

In this initial evaluation phase, the primary objective is to determine the generalization capabilities of the proposed algorithms without relying on any user-specific calibration-often referred to as *out-of-the-box performance*.

To ensure robust statistical validation and eliminate the risk of *data leakage*, a strict subject-level *K-Fold Cross-Validation* [29] protocol was implemented. In each iteration, 10% of the subjects (approximately five individuals) were completely withheld from all training and tuning phases, serving as a strict subject-level internal holdout fold. This process was repeated 10 times to ensure every participant was evaluated as part of a holdout set. Table 14 and Table 15 report the detailed clinical metrics obtained from the K=10 cross-validation, evaluated across both hemodynamic modalities: deflating (↘) and inflating (↗). The model hyperparameters were selected through manual grid search optimization on validation folds of the training set. During the deflating phase (↘), each fold utilized 552 files for training and 61 for testing. The inflating phase (↗) utilized 548 training files and 61 testing files per iteration. All four machine learning architectures exhibited lower predictive error on the ramp-up (↗) dataset under the evaluated acquisition sequence.

### 4.4. One-Point Cuff Calibration and Hybrid Personalization

In this section, we introduced personalized calibration for each user, described as a one-point cuff calibration when using a single calibration recording (N=1). We utilized the out-of-fold bias correction technique [29], which partitions the dataset identically to the *K*-fold cross-validation strategy (e.g., K=10).

In this setup, the global machine learning model is trained on the majority of the population data. During evaluation, a small subset of *N* user-specific recordings is extracted strictly for calibration (i.e., calculating a personalized pressure bias offset, without retraining the model’s weights), and the remaining files from that user are used for testing. Each calibration recording (N=1) corresponds to a single, complete finger-pressing trial containing multiple heartbeats, paired with a single reference cuff measurement, rather than a single isolated heartbeat.

To select the calibration files deterministically for each user, we perform the following steps:

- Gather all valid, preprocessed recordings for the user.
- Sort the file paths alphabetically.
- Reset the Python (3.11.9) global random seed to a fixed constant (RANDOM_SEED = 42).
- Shuffle the file paths using random.shuffle() and select the first *N* files for calibration.

Because the random seed is reset right before shuffling each user’s files, the shuffling logic remains identical across all participants (i.e., for any user with *M* files, the file permuted to index 0 is always chosen for the N=1 calibration).

**Figure 7:**
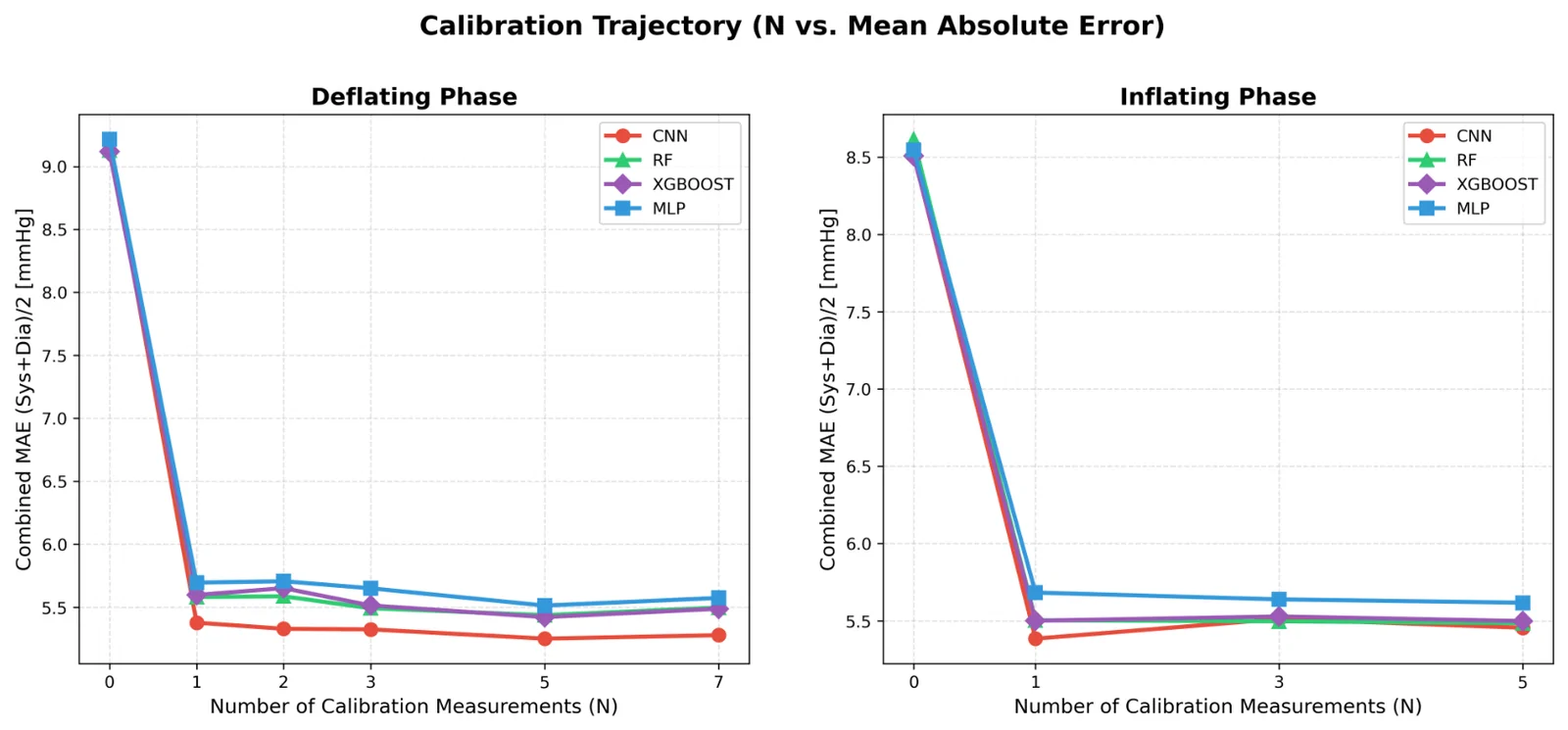
Calibration trajectory across varying numbers of localized measurements (*N*).

For each cross-validation fold, the model was trained on approximately 552 files from 90% of the participant pool (roughly 43 users). We evaluated the trajectory of improvement from minimal calibration (N=1) to extended calibration (N=3) and (N=5). As shown in Figure 7, increasing the calibration set beyond N=1 does not yield significant performance gains, indicating that a single-point calibration is sufficient to correct the bulk of the baseline shift. In Table 16 and Table 17, we highlight the results obtained for systolic and diastolic pressure using N=1.

It is important to note that all recordings for a given participant were collected within a single experimental session, and the calibration and testing data share identical physiological states (e.g., vascular tone and hydration). Thus, while this one-point calibration substantially reduces individual offset bias within the session, this protocol does not demonstrate the temporal stability of the calibration across multiple days or varying physiological conditions.

The most significant finding from the one-point cuff integration is the impact of minimal calibration. Introducing a single cuff-labeled calibration recording (N=1) yields an immediate error reduction, dropping the systolic MAE during the deflating phase from 10.42 mmHg to 5.55 mmHg (1D-CNN). This one-point calibration also reveals a nuanced physiological specialization between the hemodynamic phases. While the inflating (↗) modality dominated the out-of-the-box predictions globally, the localized calibration shifts the optimal phase depending on the target metric. The deflating (↘) phase proved slightly superior for systolic pressure estimation (5.55 mmHg MAE), whereas the inflating (↗) phase excelled significantly at diastolic estimation, dropping the MAE to an impressive 4.85 mmHg with minimal bias (+0.31 ME). This suggests that the progressive occlusion of the arterial bed during ramp-up captures the diastolic onset with higher morphological fidelity, while the controlled release during ramp-down provides cleaner systolic peaks once the baseline is anchored, while overall error metrics remain similar across architectures, the 1D-CNN exhibits a marginal predictive advantage, outperforming XGBoost by 0.2 to 0.3 mmHg and maintaining narrower standard deviations.

The objective of the N=1 paradigm is to evaluate the extent to which a single cuff-based initialization can anchor a global model to an individual’s specific baseline. However, it is important to acknowledge the potential for experimental optimism in this pilot study. Since the calibration and testing recordings were acquired within the same session under consistent physiological conditions (e.g., vascular tone, hydration levels, and ambient temperature), the observed improvement represents a within-session bias correction (resolving static inter-subject offsets) rather than genuine longitudinal calibration. While these results demonstrate that one measurement can effectively resolve inter-subject bias, future work must evaluate the temporal stability of this calibration across multiple days to satisfy the longitudinal requirements of the IEEE 1708-2025 standard [30].

### 4.5. Cross-Architectural Comparison of Ramp-Up and Ramp-Down Modalities

To evaluate if the predictive difference in the inflating (ramp-up ↗) modality is consistent across architectures rather than an artifact of specific model bias, a cross-architectural evaluation was conducted. We analyzed the minimally calibrated (N=1) diastolic mean absolute error (MAE) across four machine learning topologies: a 1D-CNN, a multi-layer perceptron (MLP), Random Forest (RF), and XGBoost.

**Figure 8:**
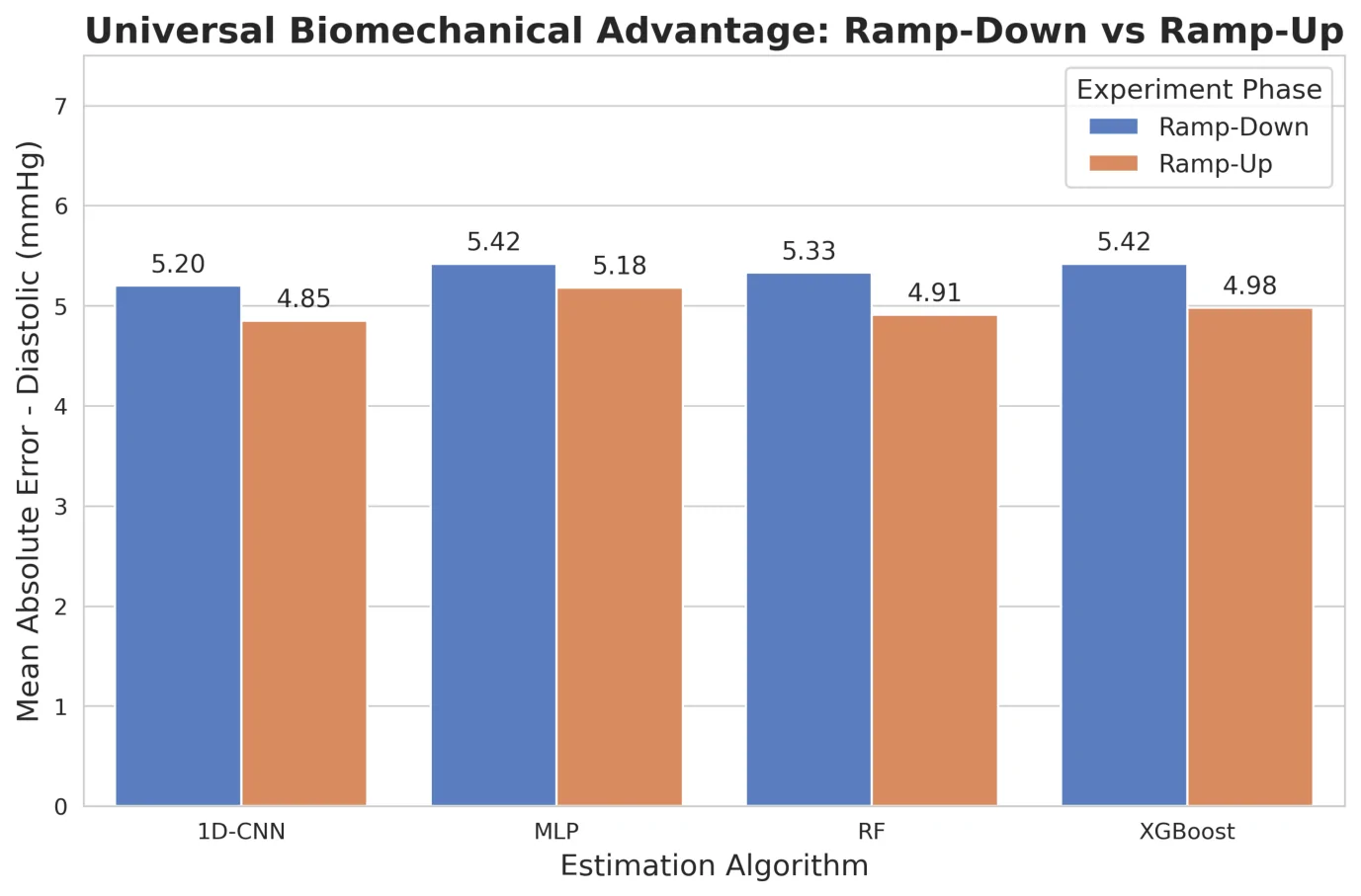
Cross-architectural comparison of diastolic mean absolute error (MAE) under optimal minimal calibration (N=1). The consistent reduction in predictive error indicates that lower estimation error for the ramp-up protocol is observed across different algorithms under the current acquisition sequence.

As illustrated in Figure 8, the ramp-up modality yielded consistent predictive error reductions across all evaluated algorithms: 8.1% for XGBoost (from 5.42 to 4.98 mmHg), 7.9% for Random Forest (from 5.33 to 4.91 mmHg), 6.7% for the 1D-CNN (from 5.20 to 4.85 mmHg), and 4.4% for the MLP (from 5.42 to 5.18 mmHg).

This consistent improvement suggests that the ramp-up (↗) trend is model-agnostic. A plausible explanation is that applying increasing pressure may help stabilize the finger–device contact, leading to fewer motion artifacts. Consequently, the machine learning algorithms receive more stable signal features, leading to consistently lower estimation errors.

### 4.6. Comparative Analysis-Global Generalization vs. One-Point Cuff Calibration

**Figure 9:**
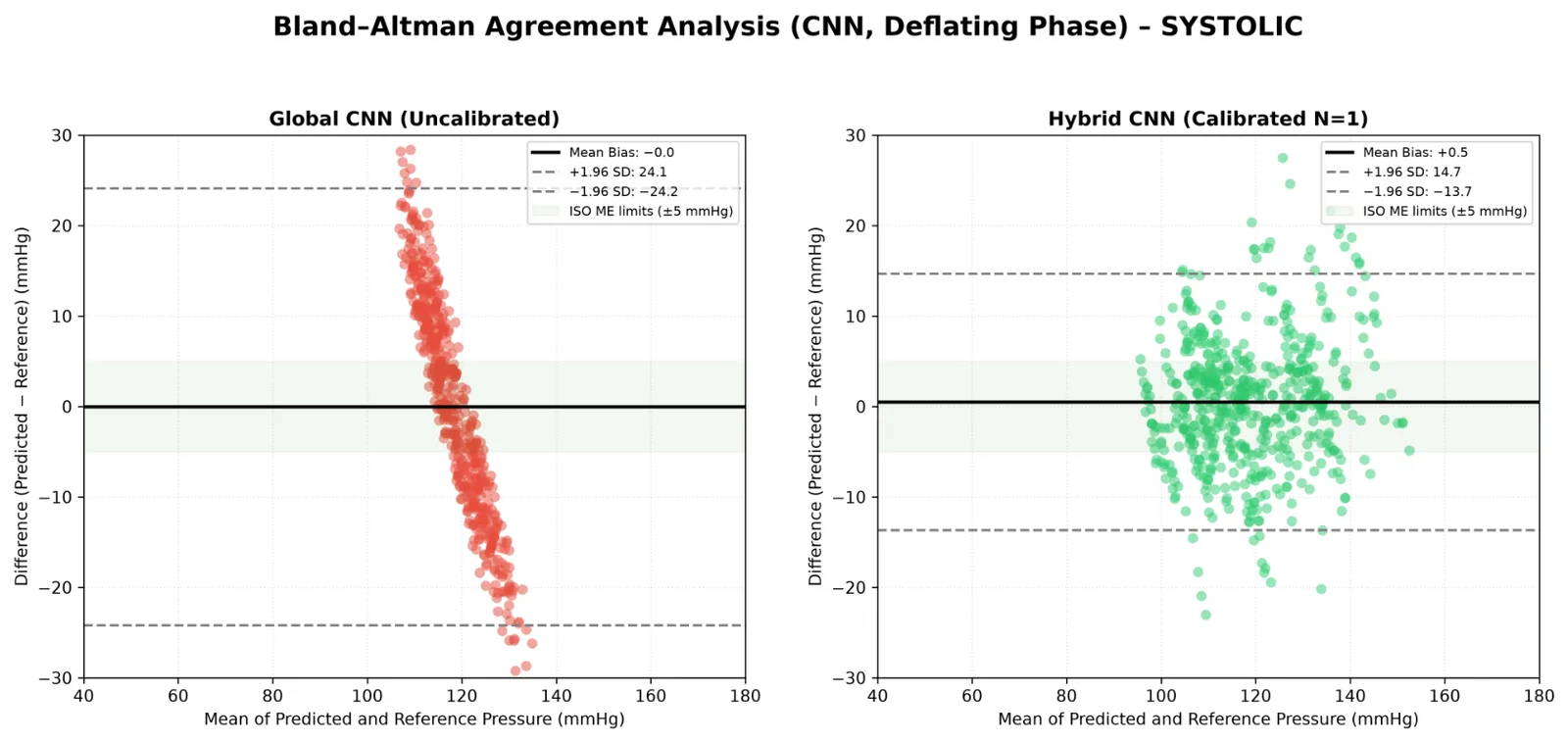
Deflating (↘) phase–systolic pressure–Bland–Altman agreement analysis.

**Figure 10:**
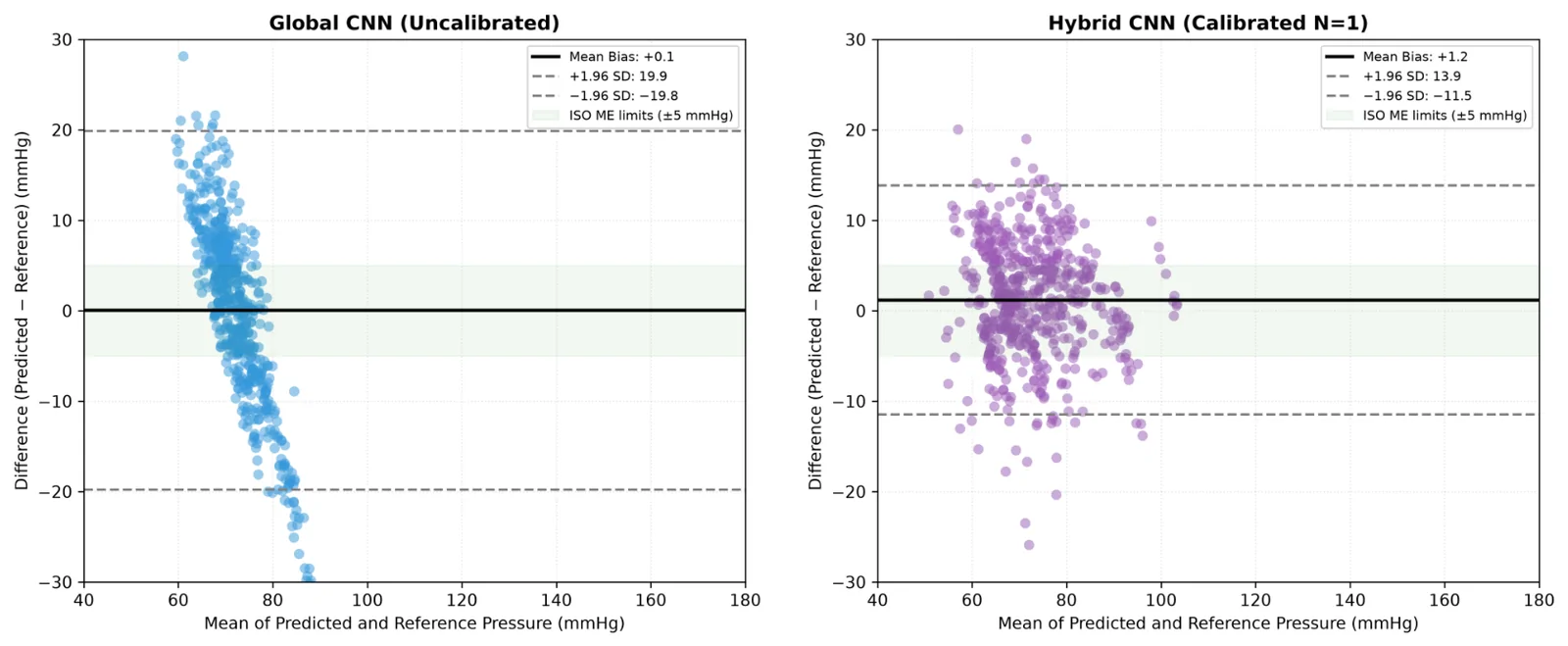
Deflating (↘) phase–diastolic pressure–Bland–Altman agreement analysis.

To visually quantify this improvement, Bland–Altman agreement analysis was conducted, comparing the uncalibrated Global CNN against the optimal one-point calibrated CNN (N=1). Figure 9 and Figure 10 illustrate this comparison for the deflating (↘) phase. We have observed a similar trend during the inflation (↗) phase as well.

In the left panels (Global CNN), the predictions exhibit a pronounced proportional bias characterized by a steep negative slope. This structural artifact indicates that without a personalized baseline, the global model overestimates hypotensive values and underestimate hypertensive ones. Conversely, the right panels (Hybrid CNN) demonstrate that a single calibration file substantially reduces this proportional bias. The predictions transition into a horizontally distributed, homoscedastic cloud centered tightly around the zero-error line.

As detailed in the statistical methodology, because the 1274 recordings are repeated measurements from 48 participants, the localized one-point calibration (N=1) minimizes the systematic inter-subject offset bias. Consequently, the inter-subject variance component is minimized, ensuring that the standard pooled calculation of the limits of agreement (LoAs) converges to the repeated-measures formulation [27] and remains statistically robust for the visual representations in Figure 9 and Figure 10.

#### Correlation Analysis: Biomechanical Instability vs. Predictive Error

To investigate whether user-induced biomechanical instability systematically degrades blood pressure estimation accuracy, a correlation analysis was performed. We evaluated the spatial variance—quantified by the combined center of pressure (CoP) mean absolute deviation (MAD)—against the absolute predictive error of the optimal Hybrid 1D-CNN model (N=1) across all 1274 clinically accepted recordings.

The analysis yielded a Pearson coefficient of r=−0.0238 (p=0.42) for systolic error and r=−0.0056 (p=0.85) for diastolic error. Because both *p*-values exceed the standard 0.05 significance threshold, and the *r*-values approach zero, the data do not indicate a statistically significant linear correlation between finger spatial instability and predictive error within the bounds of the filtered dataset.

This statistical independence suggests that the proposed architecture is relatively robust to normal contact-pressure shifts, provided the recordings pass the initial signal quality index (SQI) threshold.

### 4.7. Numerical Benchmarking Against BP Validation Thresholds

We benchmarked our optimal configuration, Hybrid 1D-CNN (N=1), against the numerical error thresholds defined by the three primary international standards for non-invasive blood pressure monitoring to evaluate its potential for future clinical applications. We emphasize that these comparisons serve strictly as preliminary numerical benchmarking to assess algorithmic feasibility in a controlled pilot cohort, and do not constitute or imply formal clinical validation, medical device compliance, or generalizability to broader clinical populations.

1. **AAMI/ISO 81060-2 Standard**
  - **Criteria:** Mean error (ME) ≤±5.0 mmHg and standard deviation (SD) ≤8.0 mmHg according to the ISO 81060-2 standard [31], which is referenced here strictly as a numerical performance benchmark rather than a direct clinical validation protocol for cuffless devices.
  - **Our Results (Deflating ↘):** Systolic ME is +0.50±7.25 mmHg; diastolic ME is +1.19±6.47 mmHg.
  - **Our Results (Inflating ↗):** Systolic ME is −1.28±7.44 mmHg; diastolic ME is +0.31±6.46 mmHg.
  - **Conclusions:** As a research prototype evaluated in a pilot study, the obtained values fall within range limits comparable to the numerical thresholds specified in the AAMI/ISO standard, though this does not constitute formal compliance. The N=1 calibration substantially reduces systematic bias, demonstrating the accuracy potential of this research system across the physiological range, though formal medical device validation requires a larger standards-compliant population.
2. **British Hypertension Society (BHS) Grading**
  - **Criteria:** Evaluates the percentage of errors ≤5, ≤10, and ≤15 mmHg. Grade A requires 60%/85%/95%. Grade B requires 50%/75%/90%.
  - **Our Results (Deflating ↘):** Systolic achieved 56%/84%/95%. Diastolic achieved 55%/88%/98%.
  - **Our Results (Inflating ↗):** Systolic achieved 50%/82%/95%. Diastolic achieved **62%/90%/97%**.
  - **Conclusions:** Under these pilot conditions, the percentage of errors falling within the 5, 10, and 15 mmHg intervals is comparable to the benchmarks referenced in the BHS protocol. Specifically, under this pilot evaluation, the inflating phase yields a lower error distribution for diastolic estimation, aligning with the numerical criteria set for the highest performance tier, while the deflating phase yields performance comparable to the secondary tier thresholds for both pressure parameters.
3. **IEEE 1708-2025 Standard (Cuffless Devices)**
  - **Criteria:** Provides a framework for the objective evaluation of cuffless BP devices, including accuracy across diverse populations and the temporal stability of the calibration.
  - **Our Results:** The N=1 calibration immediately drops the MAE by nearly 40%, compared to the global baseline. Our hardware drift analysis confirms limited zero-load baseline drift (−0.29%) over 50 days.
  - **Conclusions:** As a pre-clinical research tool, the stability and accuracy indicators align with benchmarks relevant for a pilot study. The hardware drift analysis supports the mechanical stability of the prototype, but the system is not yet a clinically validated medical device.

## 5. Discussion

### 5.1. Limitations of the Study

Several limitations must be acknowledged. First, the study population consists exclusively of healthy, normotensive young adults (mean age 20.7 years) with Fitzpatrick skin types I–II (Table 6), while this homogeneous demographic provides a highly controlled baseline for initial sensor validation, the results cannot be generalized to older populations, individuals with darker skin tones (Fitzpatrick types III–VI), or patients with chronic hypertension, arterial stiffening, or other cardiovascular pathologies. Consequently, this demographic homogeneity limits external validity, requiring all comparisons against standard error thresholds to be interpreted strictly as preliminary numerical benchmarking rather than evidence of clinical efficacy.

Second, the evaluation of the one-point cuff calibration (N=1) is restricted to a single experimental session per participant, while this single-session paradigm effectively demonstrates the feasibility of removing static inter-subject physiological bias in a controlled setting, it cannot establish genuine longitudinal calibration stability across multiple days, during which variations in vascular compliance, hydration, caffeine intake, and circadian rhythms occur. The observed error reduction must therefore be understood as a within-session baseline correction rather than a validated multi-day personalization strategy.

Third, the experimental protocol utilized a fixed acquisition order, in which all ramp-down trials were consistently executed prior to ramp-up trials. This fixed sequence introduces potential order-effect confounding: the lower estimation error and lower CoP deviation observed during ramp-up may be partially attributable to user learning, familiarization with the real-time visual biofeedback interface, or neuromuscular adaptation over time, rather than solely an intrinsic biomechanical advantage of the inflationary protocol. The rest interval of 15–30 s, while necessary to prevent prolonged venous pooling, is relatively short and could permit minor carry-over effects from muscle fatigue. Future validation protocols must incorporate a randomized or counterbalanced trial order across subjects to eliminate sequence bias and substantiate a causal biomechanical interpretation.

### 5.2. Future Research Directions

While the real-time visual CoP feedback is designed to guide the user, this study does not include a direct comparison with a group of participants performing measurements without visual feedback. Such an analysis to quantify the impact of visual biofeedback remains a topic for future research.

Although the ISO 81060-2 standard is designed for intermittent automated cuff-based sphygmomanometers, its numerical error limits serve as a useful benchmark. Future work will focus on scaling the participant pool to N≥85 to satisfy clinical sample-size recommendations, even though formal validation of cuffless devices requires dedicated protocols such as the IEEE 1708-2025 standard.

In addition to the cohort size, a constraint of the current hardware is that the conversion from measured force (grams) to estimated contact pressure (mmHg) uses a fixed effective contact area of 1.45 cm^2^, derived from established fingertip morphometry literature [14], while this constant serves as a reliable baseline for a pilot study, future iterations will incorporate individual-specific contact area estimations to account for variance in finger size and tissue elasticity.

## 6. Conclusions

This paper presented the design, implementation, and evaluation of a novel cuffless finger blood pressure monitor, addressing the critical need for accessible and non-invasive cardiovascular monitoring. By integrating a three-point force-sensing array with a PPG sensor, we addressed a primary limitation of conventional optical BP devices: the lack of precise contact pressure quantification. We conducted an evaluation, demonstrating that our hybrid machine learning architectures can generate intermittent or spot-check blood pressure estimations that are comparable to the numerical accuracy benchmarks of international medical device standards in a pre-clinical pilot cohort.

In this pre-clinical pilot dataset, the calibrated model falls within the numerical mean-error and standard-deviation thresholds commonly used in AAMI/ISO-style evaluation, but the study does not constitute formal clinical validation. It is important to contextualize these results as a pre-clinical pilot validation study. The current experimental group comprises 48 subjects, which provides statistical indications of the algorithmic feasibility, the baseline adjustment effect of N=1 calibration, and hardware stability under pilot conditions.

## Figures and Tables

**Table 1:** State of the art vs. proposed method.

Design Aspect	Common Lit. Approach	Our Proposed Solution
Force Sensing Reasoning	Single load cell (one-axis) [4] Simple, but prone to tilting errors.	Three-point array (three sensors) Isostatic stability; detects off-center presses.
Alignment Reasoning	Physical/passive guide [5] Assumes centering; no error correction.	Visual CoP feedback Real-time Bullseye ensures geometric centering.
Protocols Reasoning	Uncontrolled pressure [6] Variable rates distort the envelope.	Guided ramp-up ↗ & ramp-down ↘ Visual pacing ensures linear pressure application.
Calibration Reasoning	Standard linear [4] Ignores sensor nonlinearity.	Hybrid Ridge Regression Compensates for drift and non-linear response.

*Note:* ↗ indicates the inflationary (ramp-up) modality with gradual pressure increase and ↘ indicates the deflationary (ramp-down) modality with gradual pressure release.

**Table 2:** Comparison of regression architectures (centered training fitting error).

Model Architecture	MAE (g) ↓	Stability
Linear (Standard OLS)	**0.85**	Low (Off-Center Drift)
Linear Ridge	1.60	**High**
Quadratic (Polynomial)	2.08	Low (Overfitting)
Cubic (Polynomial)	1.64	Low (Overfitting)
Quadratic Ridge (Independent)	0.87	Low (Overfitting)

*Note:* ↓ indicates that lower values denote better performance. Bold indicates the best value per metric.

**Table 3:** Evaluation of the hybrid force calibration model (centered training fitting error).

Processing Phase	Model Architecture	MAE (g) ↓	Stability
Baseline	Linear (Standard OLS)	**0.85**	Low
Stage 1	Linear Ridge	1.60	High
**Final Arch.**	**Hybrid (Linear Ridge + Quadratic Correction)**	1.20	**High**

*Note:* ↓ indicates that lower values denote better performance. Bold indicates the selected model and lowest error/highest stability.

**Table 4:** Independent test performance: spatial generalization on the 9-point validation grid (200 g reference load).

Model Architecture	MAE (g) ↓
**Hybrid (Linear Ridge + Quadratic Correction)**	**4.72**
Linear Ridge	4.93
Quadratic Ridge (Independent)	28.18
Linear (Standard OLS)	32.96
Cubic (Polynomial)	49.15
Quadratic (Polynomial)	53.72

*Note:* ↓ indicates that lower values denote better performance. Bold denotes the best-performing model.

**Table 5:** Overview of the data collection phases.

Phase	Objective and Actuation Method	Reference	Reps	Rest Int.
1. Calibration	Arm Dominance ($\Delta_{L-R}$): simultaneous dual-cuff inflation. Cuffs are swapped to remove bias.	Dual Cuffs	3	2 min
2. Ramp-Down ↘	Deflationary: rapid press to target (180 mmHg), followed by visually guided slow release.	Reference Arm	10–15	15–30 s
3. Ramp-Up ↗	Inflationary: visually guided, gradual pressure increase from baseline to target (180 mmHg).	Reference Arm	10–15	15–30 s

*Note:* ↗ indicates the inflationary (ramp-up) modality with gradual pressure increase and ↘ indicates the deflationary (ramp-down) modality with gradual pressure release.

**Table 6:** Baseline characteristics of the study population and dataset hemodynamic coverage.

Characteristic	Male (n=26)	Female (n=22)	Total N=48
Demographics			
Age (years)	20.6±0.6	20.7±0.8	20.7±0.7
Range (years)	20–22	19–22	19–22
Smoking Status (n,%)	2 (7.7%)	3 (13.6%)	5 (10.4%)
Physical Metrics			
Height (cm)	179.4±7.2	168.5±5.3	174.4±8.4
Weight (kg)	72.5±12.5	58.0±7.1	65.9±12.6
BMI (${\mathrm{kg/m}}^{2}$)	22.4±2.8	20.4±1.9	21.5±2.6
Optical & Morphometry			
Finger Width (mm)	16.0±1.2	14.7±1.2	15.4±1.4
Skin Type: Type 1 (n,%)	3 (11.5%)	5 (22.7%)	8 (16.7%)
Skin Type: Type 2 (n,%)	23 (88.5%)	17 (77.3%)	40 (83.3%)
Hemodynamic Range			
Systolic BP (mmHg)	88–150	87–155	87–155
Diastolic BP (mmHg)	43–98	48–103	43–103
Heart Rate (bpm)	50–107	51–129	50–129

*Note:* Values are reported as mean ± standard deviation or count (percentage). *N* denotes the total cohort size (N=48), whereas *n* denotes subgroup sample sizes or category frequency counts. Hemodynamic parameters represent post-filtered, clean data yields.

**Table 7:** Distribution of dataset telemetry across blood pressure range bins (N=1274 clean recordings).

Systolic Range (mmHg)	Count (n,%)	Diastolic Range (mmHg)	Count (n,%)
<100	110 (8.6%)	<60	185 (14.5%)
100–120	720 (56.5%)	60–80	893 (70.1%)
120–140	411 (32.3%)	80–90	140 (11.0%)
140–160	33 (2.6%)	90–100	47 (3.7%)
>160	0 (0.0%)	>100	9 (0.7%)

**Table 8:** Summary of signal quality index (SQI) exclusion criteria and protocol yields.

Exclusion Criterion	Technical Rationale	Ramp-Down	Ramp-Up	Total (*N*)
High-Motion Artifacts	Guidance MAD >15.0 mmHg	3	2	5
Poor Monotonicity	Correlation |r|<0.90 with target	2	1	3
Insufficient Pulse Count	<8 valid pulses detected	3	2	5
Physiological Out-of-Bounds	Out of physiological BP range	2	2	4
Total Excluded	1.3% of initial recordings	10	7	17
Retained Yield	98.7% of initial recordings	637	637	1274

**Table 9:** Granular biomechanical stability: mean absolute deviation (MAD) for OX and OY axes.

Hemodynamic Phase	OX MAD (a.u.) ↓	OY MAD (a.u.) ↓	Combined Error (a.u.) ↓
Ramp-Down	0.162±0.131	0.313±0.225	0.381±0.216
Ramp-Up	0.155±0.140	0.243±0.211	0.317±0.215

*Note:* Values are reported as mean absolute deviation (MAD) ± standard deviation. Combined error represents the Euclidean distance ($\sqrt{\Delta x^{2}+\Delta y^{2}}$) from the optimal geometric target center. ↓ indicates that lower values denote better stability. Bold denotes the lower deviation.

**Table 10:** Longitudinal sensor drift summary (cumulative baseline analysis).

Metric	Value
Total Study Duration	50 Days
Initial Baseline (Total Counts)	12,017.7
Final Baseline (Total Counts)	11,982.5
Absolute Net Drift	−35.2
Percentage Net Drift	−0.29%

*Note:* Baselines are calculated as the sum of p1_zero, p2_zero, and p3_zero.

**Table 11:** Description of the 17 hand-crafted pulse-level features.

Feature Name	Physical/Morphological Description
mean_p	Mean applied pressure during the heartbeat segment (mmHg)
norm_amp	Normalized pulse amplitude computed as $(AC_{\mathrm{peak-to-peak}}/DC_{\mathrm{avg}})\times 1000$ (IR channel)
red_amp	Normalized pulse amplitude of the red channel $\left(AC_{\mathrm{peak-to-peak}}/DC_{\mathrm{avg}}\right)$
pulse_width	Total duration of the heartbeat segment (number of samples)
force_var	Standard deviation of the raw force sensor readings ($p_{1},p_{2},p_{3}$) during the pulse
skew	Skewness of the filtered IR waveform, capturing asymmetry of the pulse
kurtosis	Kurtosis of the filtered IR waveform, describing the peak sharpness
auc	Area under the baseline-subtracted filtered IR waveform
pulse_corr	Pearson correlation coefficient between the filtered IR and red waveform segments
imbalance	Spatial pressure deviation computed as $SD\left(p_{1},p_{2},p_{3}\right)/Mean\left(p_{1},p_{2},p_{3}\right)$
pressure_trend	Change in applied pressure during the pulse ($P_{\mathrm{end}}-P_{\mathrm{start}}$)
systolic_rise_time	Duration of the systolic rise phase (samples from start to peak)
diastolic_decay_time	Duration of the diastolic decay phase (samples from peak to end)
crest_time_ratio	Ratio of systolic rise time to the total pulse width
amp_pressure_ratio	Ratio of normalized amplitude to the mean applied pressure
vpg_max_slope	Maximum slope of the velocity photoplethysmogram (first derivative, VPG)
apg_b_a_ratio	Absolute ratio of minimum to maximum acceleration peaks (second derivative, APG)

**Table 12:** Hyperparameter configurations for the classical machine learning models.

Model Architecture	Hyperparameter/Parameters	Value/Configuration
XGBoost	Number of Gradient-Boosted Trees (n_estimators)	150
	Learning Rate/Shrinkage (learning_rate)	0.08
	Maximum Tree Depth (max_depth)	5
	Random State Seed	42
Random Forest	Number of Trees (n_estimators)	100
	Split Criterion	Mean Squared Error (MSE)
	Minimum Samples per Leaf	1 (Default)
	Random State Seed	42
MLP Regressor	Network Topology (Hidden Layers)	Two layers: 64 and 32 units
	Activation Function	Rectified Linear Unit (ReLU)
	Solver/Optimizer	Adam
	Maximum Iterations (max_iter)	500 epochs
	Feature Normalization	Standard Scaler (*Z*-score)
	Random State Seed	42

**Table 13:** 1D-CNN architecture details and hyperparameters.

Component	Specification	Detail/Output Shape
Inputs	Dual-Input	Waveform (128×1), Pressure (1×1)
Layer 1	Convolutional (1D)	16 filters, Kernel: 5, Act: ReLU
Layer 2	Max Pooling (1D)	Pool Size: 2
Layer 3	Convolutional (1D)	32 filters, Kernel: 3, Act: ReLU
Layer 4	Max Pooling (1D)	Pool Size: 2
Layer 5	Flattening	Output: 960 units
Fusion	Concatenation	Temporal (960) + Pressure (1)
Layer 6	Dense (Fully Connected)	32 units, Act: ReLU
Layer 7	Output Layer	1 unit, Act: Linear
Optimizer	Adam	Learning Rate: 0.001 (Default)
Loss Function	Mean Absolute Error	Evaluated in mmHg
Training	Configuration	Epochs: 10, Batch Size: 64
Complexity	Total Trainable Params	≈32,481 Parameters

**Table 14:** Universal generalization of out-of-the-box performance for systolic pressure via 10-fold cross-validation.

Model Architecture	MAE ↓	ME ± SD	≤5/10/15 mmHg
Phase: Deflating (Ramp-Down)			
1D-CNN	10.42	+0.37±10.54	27/52/76%
MLP	10.51	+1.71±10.82	26/54/75%
Random Forest	10.57	+0.78±10.82	25/52/75%
XGBoost	10.46	+0.66±10.78	25/52/76%
Phase: Inflating (Ramp-Up)			
1D-CNN	**9.44**	+1.02±9.60	29/**61**/79%
MLP	9.71	+1.83±10.17	29/57/79%
Random Forest	9.50	+0.75±9.93	29/59/80%
XGBoost	9.46	+0.50±9.89	**30**/60/**81**%

*Note:* Bold indicates the lowest MAE/best result.

**Table 15:** Universal generalization of out-of-the-box performance for diastolic pressure via 10-fold cross-validation.

Model Architecture	MAE ↓	ME ± SD	≤5/10/15 mmHg
Phase: Deflating (Ramp-Down)			
1D-CNN	7.98	+0.13±9.01	38/71/87%
MLP	7.92	+1.17±9.06	37/69/87%
Random Forest	7.69	+0.21±9.09	**42**/**72**/88%
XGBoost	7.78	+0.15±9.15	40/70/88%
Phase: Inflating (Ramp-Up)			
1D-CNN	7.60	+0.38±8.34	37/68/**90**%
MLP	**7.39**	+0.85±8.20	41/**72**/**90**%
Random Forest	7.73	+0.24±8.42	36/69/88%
XGBoost	7.56	+0.06±8.27	37/69/**90**%

*Note:* Bold indicates the lowest MAE/best result.

**Table 16:** One-point cuff calibration performance for systolic pressure using a single calibration file (N=1).

Model Architecture	MAE ↓	ME ± SD	≤5/10/15 mmHg
Phase: Deflating (Ramp-Down)			
1D-CNN	5.55	+0.50±7.25	**56**/84/95%
MLP	5.97	+0.89±7.65	52/81/94%
Random Forest	5.83	+0.52±7.55	52/81/95%
XGBoost	5.78	+0.68±7.47	53/83/95%
Phase: Inflating (Ramp-Up)			
1D-CNN	5.92	−1.28±7.44	50/82/95%
MLP	6.18	−0.83±7.82	48/82/94%
Random Forest	6.10	−0.75±7.72	50/80/95%
XGBoost	6.02	−1.01±7.59	49/81/95%

*Note:* Bold indicates the lowest MAE/best result.

**Table 17:** One-point cuff calibration performance for diastolic pressure using a single calibration file (N=1).

Model Architecture	MAE ↓	ME ± SD	≤5/10/15 mmHg
Phase: Deflating (Ramp-Down)			
1D-CNN	5.20	+1.19±6.47	55/88/98%
MLP	5.42	+1.03±6.80	53/86/97%
Random Forest	5.33	+1.09±6.68	54/86/97%
XGBoost	5.42	+1.03±6.71	53/86/98%
Phase: Inflating (Ramp-Up)			
1D-CNN	**4.85**	+0.31±6.46	**62/90/97%**
MLP	5.18	+0.63±6.71	59/86/97%
Random Forest	4.91	+0.55±6.48	62/89/97%
XGBoost	4.98	+0.52±6.55	62/88/97%

*Note:* Bold indicates the lowest MAE/best result.

## Data Availability

The original data presented in the study are openly available in Zenodo under a Creative Commons Attribution 4.0 International (CC BY 4.0) license at https://doi.org/10.5281/zenodo.21490890, reference [32].

## References

[1] World Health Organization (2025). Cardiovascular Diseases (CVDs) Key Facts. https://www.who.int/news-room/fact-sheets/detail/cardiovascular-diseases-(cvds).

[2] Gosse P., Bougaleb M., Egloff P., Lemetayer P., Clementy J. (1994). Clinical significance of white-coat hypertension. J. Hypertens..

[3] Andreozzi E., Sabbadini R., Centracchio J., Bifulco P., Irace A., Breglio G., Riccio M. (2022). Multimodal finger pulse wave sensing: Comparison of forcecardiography and photoplethysmography sensors. Sensors.

[4] Chandrasekhar A., Kim C.S., Naji M., Natarajan K., Hahn J.O., Mukkamala R. (2018). Smartphone-based blood pressure monitoring via the oscillometric finger-pressing method. Sci. Transl. Med..

[5] Chandrasekhar A., Yavarimanesh M., Natarajan K., Hahn J.O., Mukkamala R. (2020). PPG sensor contact pressure should be taken into account for cuff-less blood pressure measurement. IEEE Trans. Biomed. Eng..

[6] Freithaler M., Chandrasekhar A., Dhamotharan V., Landry C., Shroff S.G., Mukkamala R. (2023). Smartphone-Based Blood Pressure Monitoring via the Oscillometric Finger Pressing Method: Analysis of Oscillation Width Variations Can Improve Diastolic Pressure Computation. IEEE Trans. Biomed. Eng..

[7] Gul F.A.I., Tudose D., Ţurcanu T. A versatile IoT development board for environmental sensing and biometric applications. Proceedings of the 23rd RoEduNet Conference: Networking in Education and Research (RoEduNet).

[8] Autodesk Inc. (2024). Autodesk Fusion 360. https://www.autodesk.com/products/fusion-360/overview.

[9] Honeywell International Inc. FMAMSDXX015WCSC3 Force Sensor Datasheet. https://prod-edam.honeywell.com/content/dam/honeywell-edam/sps/siot/ja/products/sensors/force-sensors/microforce-fma-series/documents/hon-ia-hss-force-fma-series-datasheet-32347833-ciid-181799.pdf.

[10] Analog Devices Inc. (2018). MAX30102: High-Sensitivity Pulse Oximeter and Heart-Rate Sensor for Wearable Health. Datasheet. https://www.analog.com/media/en/technical-documentation/data-sheets/max30102.pdf.

[11] NXP Semiconductors (2021). UM10204: I2C-Bus Specification and User Manual. Rev. 7.0. https://www.nxp.com/docs/en/user-guide/UM10204.pdf.

[12] Motorola Inc. (2000). SPI Block Guide V03.06.

[13] Thompson A., Taylor B.N. (2008). Guide for the Use of the International System of Units (SI).

[14] Dandekar K., Raju B.I., Srinivasan M.A. (2003). 3-D finite-element models of human and monkey fingertips to investigate the mechanics of tactile sense. J. Biomech. Eng..

[15] Willmott C.J., Matsuura K. (2005). Advantages of the Mean Absolute Error (MAE) over the Root Mean Square Error (RMSE) in Assessing Average Model Performance. Clim. Res..

[16] Hoerl A.E., Kennard R.W. (1970). Ridge regression: Biased estimation for nonorthogonal problems. Technometrics.

[17] Wilcoxon F. (1945). Individual comparisons by ranking methods. Biom. Bull..

[18] Cohen J. (1988). Statistical Power Analysis for the Behavioral Sciences.

[19] Elgendi M. (2012). On the analysis of fingertip photoplethysmogram signals. Curr. Cardiol. Rev..

[20] Drzewiecki G., Hood R., Apple H. (1994). Theory of the oscillometric maximum and the systolic and diastolic detection ratios. Ann. Biomed. Eng..

[21] Allen J. (2007). Photoplethysmography and its application in clinical physiological measurement. Physiol. Meas..

[22] Geddes L.A., Voelz M., Combs C., Reiner D., Babbs C.F. (1982). Characterization of the oscillometric method for measuring indirect blood pressure. Ann. Biomed. Eng..

[23] Chen T., Guestrin C. XGBoost: A scalable tree boosting system. Proceedings of the 22nd ACM SIGKDD International Conference on Knowledge Discovery and Data Mining.

[24] Breiman L. (2001). Random forests. Mach. Learn..

[25] Rumelhart D.E., Hinton G.E., Williams R.J. (1986). Learning representations by back-propagating errors. Nature.

[26] LeCun Y., Bottou L., Bengio Y., Haffner P. (1998). Gradient-based learning applied to document recognition. Proc. IEEE.

[27] Bland J.M., Altman D.G. (1999). Measuring agreement in method comparison studies. Stat. Methods Med. Res..

[28] Shapiro S.S., Wilk M.B. (1965). An analysis of variance test for normality (complete samples). Biometrika.

[29] Kohavi R. A study of cross-validation and bootstrap for accuracy estimation and model selection. Proceedings of the 14th International Joint Conference on Artificial Intelligence (IJCAI).

[30] (2026). IEEE Standard for Wearable, Cuffless Blood Pressure Measuring Devices.

[31] (2018). Non-Invasive Sphygmomanometers—Part 2: Clinical Investigation of Intermittent Automated Measurement Type.

[32] Gul F.A.I., Tudose D. (2026). Dataset for ‘A Center-of-Pressure Guided Finger-Press Sensor for Cuffless Blood Pressure Estimation’. Zenodo. https://zenodo.org/records/21490890.

